# Pharmacomicrobiology of Methotrexate in Rheumatoid Arthritis: Gut Microbiome as Predictor of Therapeutic Response

**DOI:** 10.3389/fimmu.2021.789334

**Published:** 2021-12-16

**Authors:** Huanhuan Yan, Rui Su, Hongwei Xue, Chong Gao, Xiaofeng Li, Caihong Wang

**Affiliations:** ^1^ Department of Rheumatology, The Second Hospital of Shanxi Medical University, Taiyuan, China; ^2^ Pathology, Joint Program in Transfusion Medicine, Brigham and Women’s Hospital/Children’ s Hospital, Harvard Medical School, Boston, MA, United States

**Keywords:** methotrexate (MTX), rheumatoid arthritis (RA), gut microbiome, pharmacomicrobiology, precision medicine

## Abstract

Rheumatoid arthritis (RA) is a disabling autoimmune disease with invasive arthritis as the main manifestation and synovitis as the basic pathological change, which can cause progressive destruction of articular cartilage and bone, ultimately leading to joint deformity and loss of function. Since its introduction in the 1980s and its widespread use in the treatment of RA, low-dose methotrexate (MTX) therapy has dramatically changed the course and outcome of RA treatment. The clinical use of this drug will be more rational with a better understanding of the pharmacology, anti-inflammatory mechanisms of action and adverse reaction about it. At present, the current clinical status of newly diagnosed RA is that MTX is initiated first regardless of the patients’ suitability. But up to 50% of patients could not reach adequate clinical efficacy or have severe adverse events. Prior to drug initiation, a prognostic tool for treatment response is lacking, which is thought to be the most important cause of the situation. A growing body of studies have shown that differences in microbial metagenomes (including bacterial strains, genes, enzymes, proteins and/or metabolites) in the gastrointestinal tract of RA patients may at least partially determine their bioavailability and/or subsequent response to MTX. Based on this, some researchers established a random forest model to predict whether different RA patients (with different gut microbiome) would respond to MTX. Of course, MTX, in turn, alters the gut microbiome in a dose-dependent manner. The interaction between drugs and microorganisms is called pharmacomicrobiology. Then, the concept of precision medicine has been raised. In this view, we summarize the characteristics and anti-inflammatory mechanisms of MTX and highlight the interaction between gut microbiome and MTX aiming to find the optimal treatment for patients according to individual differences and discuss the application and prospect of precision medicine.

## 1 Introduction

Rheumatoid arthritis (RA) is a systemic autoimmune disease characterized by persistent synovitis, chronic and aggressive arthritis. Although the treatment of RA has made great progress in recent years, the overall remission rate of RA is still not optimistic, and it is almost impossible to reverse once bone or joint destruction occurs (1, 2). The pathogenesis of RA is still not fully understood, which may be caused by the complex interaction between genetic environment and immune factors, resulting in immune system dysregulation and lack of autoimmune tolerance. Although several effective treatments have been developed recently, low-dose methotrexate (MTX) remains the anchor agent for RA treatment, which is generally considered to enhance the efficacy of most biologics in RA (3, 4). However, a large proportion of RA patients do not respond to oral MTX or have severe adverse reactions, especially gastrointestinal toxicity is common, which can be troublesome (3–6). Therefore, it is urgent to find out the possible reasons for individual differences and the biological indicators that can predict the clinical efficacy of MTX to guide clinical medication.

An adult human has about 2*10 ^14 host cells, but this is only a tenth of the bacteria that live in the mucosae of the body (the intestine, reproductive and respiratory tract) and outside the body (hair and skin).Thus, the number of microbes living in the human body is extremely huge (7). These microbial communities are intimately involved in our body’s metabolism, and some emerging research is trying to deciphering the complex cross-border communication network between our immune system and the microbial community that exists in our bodies (8). The gastrointestinal tract is home to about 1,500 species of more than 100 trillion microbes, with the highest diversity and density of microbes ever seen (8). The microbiome is involved in many physiological processes, including digestion and metabolism, and the development and regulation of the immune system. In turn, the host provides living space and nutrients for the microbes. Host-microbial interaction, especially in the gut, promotes the development and regulation of the host immune system and is essential for the stability of the entire complex microbial community (9, 10). In other word, gut microbiome is an important immune organ and plays an indispensable role in immune response and tolerance (11, 12). The gut microbiome is increasingly considered to be involved in the processing of various exogenous substances and can also influence host responses to various compounds, including all kinds of drugs. And in this process, gut microbes are evolving under the influence of exogenous compounds. Metagenomics of intestinal microorganisms plays an important role in the efficacy and toxicity of many drugs, and its changes may affect the performance of drugs (13–16). 

Now the most recent view on MTX and the gut microbiota is that gut microbiome can affect the bioavailability of MTX and some characteristics of gut microbiome can be considered as a predictor of clinical response of this drug (17). In addition, gut microbiome is associated with adverse reactions to MTX, especially gastrointestinal reactions (18). Of course, MTX also affects the amount, diversity, and major components of the gastrointestinal microbiota (18, 19). Pharmacomicrobiology, based on drug-microbe potentially complex interactions has been used in various fields (20, 21). Precision medicine based on pharmacomicrobiology, while strictly integrating clinical factors and host genomics will be the development direction of rheumatoid diseases including RA in the near future (17, 21). In this review, we focus on the pharmacology, anti-inflammatory mechanism, adverse reactions of MTX and diagnostic and predictive effect of gut microbiome on clinical efficacy of oral MTX to recognize the role of pretreatment of gut microbiome in improving clinical efficacy of MTX, in order to provide a new therapy for the targeted treatment of RA patients who fail to respond within the early therapeutic window of opportunity.

## 2 MTX and Its Mechanisms of Action in RA

**Figure 1:**
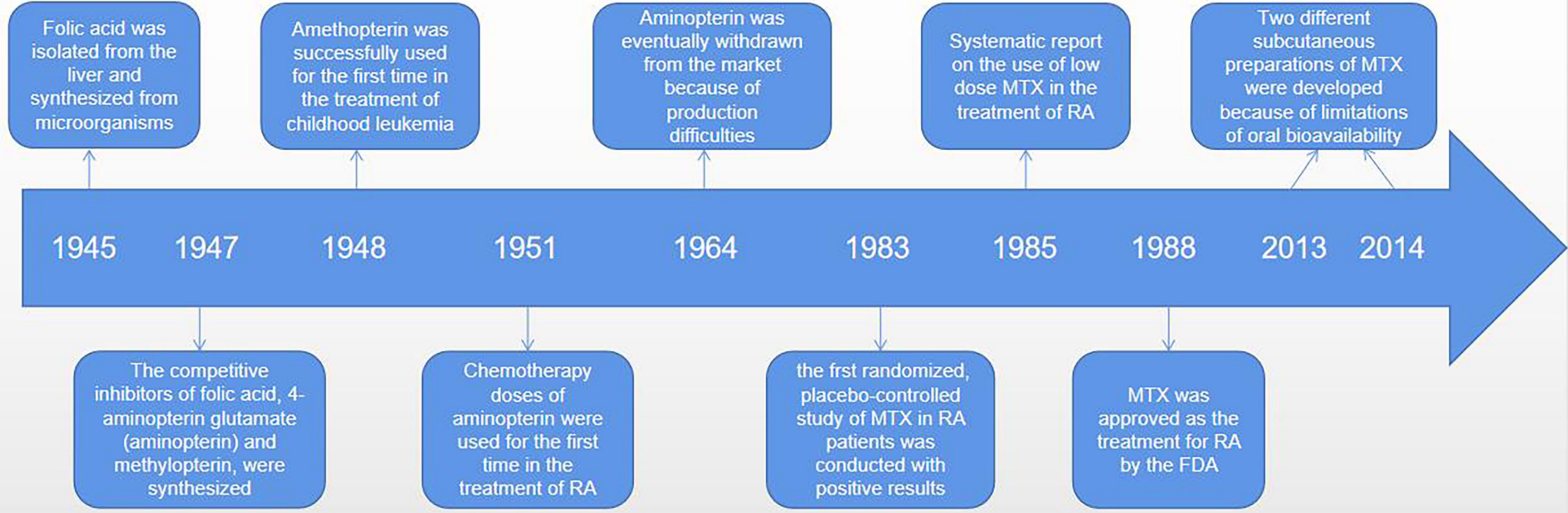
The discovery, synthesis and widespread use of MTX was a difficult process that took many decades. An American biochemist of Indian origin, called Yella Pragada Subbarow, worked with his team on isolating folic acid from liver and synthetized it from microbial source in 1945. In 1947, Yellapragada Subbarow, a biochemist at Harvard University, and his colleagues synthesized competitive inhibitors of folic (aminopterin and methylopterin) based on their structural similarity to folate and their ability to inhibit folate-dependent enzymes. Because of instability of the aminopterin, its analogue amethopterin became a popular drug. And these drugs were first used, in high doses, to treat childhood leukaemia in 1948. Amethopterin became known as MTX. And aminopterin, in chemotherapeutic doses, was first used for the treatment of RA in 1951. Due to production difficulties, MTX was eventually withdrawn from the market, leaving only MTX. In 1983, the first randomized, placebo-controlled study of MTX in patients with rheumatoid arthritis showed positive results, and MTX was approved for RA by the FDA in 1988. Since its initial approval, MTX use has increased significantly, and more RA patients have received MTX therapy. More importantly, recognizing the limitations of oral bioavailability, two different parenteral (subcutaneous) preparations were developed in 2013 and 2014.

MTX (4-amino-4-deoxy-N-10-methylpteroylglutamic acid) is an anti-folate cellular immunosuppressant. Low doses (< 15mg/per week) of MTX are widely used to treat autoimmune diseases such as RA or psoriasis (19, 22). However, higher doses of MTX (typically between 15 to 500 mg/kg) are effective in the treatment of cancer diseases, mainly for acute lymphoblastic leukemia, and osteosarcoma and lymphoma (19, 23–25). Ever since Sidney Faber discovered MTX in the 1980s, low-dose MTX is generally the first-line drug for the treatment of RA (26, 27). It is possible that MTX has been enduring because it is well tolerated, safe, and significantly less costly (28–30). It was a long and difficult journey from the initial discovery of MTX to widespread use around the world (26) (see **Figure 1**). Currently, once RA is diagnosed, rheumatologists recommend taking MTX orally. If the patient has a poor respond or strong adverse reaction to MTX, the patient is advised to continue taking it along with the biologic, since MTX appears to have an additive effect on biologic drugs (4). At low doses, MTX exhibits anti-inflammatory properties, but at high doses or for long periods of time, it may cause the pathological multi-organ toxicity, including gastrointestinal, myelotoxicity, cardiotoxicity, nephrotoxicity, and hepatotoxicity, which limits its therapeutic potential (18, 31–33). Why do different individuals respond differently to MTX? As we learn more about the pharmacological effects, anti-inflammatory mechanisms and interactions with gut microbes of MTX, the answer to this question may become more and more clear. In fact, emerging data suggest that it is possible to identify appropriate biomarkers of the gut microbiome to determine who responds best to MTX and has the fewest side effects – that is, who will benefit the most from MTX treatment.

### 2.1 Pharmacokinetics of MTX and Gut Microbiome

MTX therapy for RA is usually effective in the range of 15 to 25 mg and is usually initiated as monotherapy (22, 27). The intestinal tract has a limited ability to absorb MTX, so oral it has limited bioavailability *in vivo* (34, 35), which may be related to gut microbiota. The maximal absorption of a single oral dose is <25mg (3). Therefore, rheumatologists recommend that patients take the required dose of medication orally for one day to obtain better clinical response from MTX, rather than taking a daily dose. Due to the complex gastrointestinal toxicity of MTX, attention has shifted from oral to subcutaneous. A recent study (36) retrospectively analyzed for rheumatology clinic attendances at a large North-East England hospital and concluded when the dose of MTX in RA patients > 15 mg/week, subcutaneous method is safer than oral medicine. Specifically, the subcutaneous approach reduced the incidence of neutropenia and gastrointestinal symptoms. One problem, however, is that patients who experience adverse events with oral MTX are less likely to switch to subcutaneous treatment and are likely to experience the side effects of oral MTX again. Therefore, the search for predictors of MTX before treatment is irreplaceable.

After taking MTX orally, it is first actively absorbed by the proximal jejunum. The degree and dose of absorption are attributable to saturation of reduced folate carrier 1(RFC1) (37). The absorptivity of MTX is so high that the plasma concentration reached its maximum value within 0.75 ~ 2 h after oral Administration (26). But this drug has a relatively short half-life of about 1 hour (26)and after 18 hours its concentration in the serum is almost zero and undetectable (3). MTX enters the circulation from the gut, where 35 to 50% of the drug binds to albumin (26). Most MTX is transferred by RFC1 into cells, mainly red blood cells, white blood cells, synovial cells and liver cells. In red blood cells, MTX is polyglutamated by adding glutamate groups to the adjacent glutamate leaf terminal carboxyl group in the γ linkage (3, 26, 27). And the whole reaction is catalyzed by the enzyme folylpolyglutamyl synthetase. Red blood cells are a repository for MTX. Thus, the toxicity of MTX can also be affected by the low dose of weekly administration, which should be considered in clinical use of this drug. In the liver, MTX is oxidized to 7-hydroxyl MTX (7-OH-MTX) by aldehyde oxidase (AO), and then both parent compounds and metabolites mixed into the bile which then passes though the common bile duct and into the intestine, to duodenum, to be precise (26, 38, 39). Here, they can undergo further metabolism and biotransformation through the gut microbiome (19, 40, 41). Drugs and metabolites that enter the intestinal tract can return to the liver *via* the portal vein, which is known as the enterohepatic circulation. Cholates (unbound bile salts) can inhibit this circulation, which is very important to mitigate potential adverse events and has been showed to reduce the potential toxicity of MTX (42, 43). MTX and its metabolites are mainly excreted in urine (26, 27, 44), up to 30% are metabolized in bile (26, 45) however, only 1-2% are excreted in feces (26, 35). 

**Figure 2:**
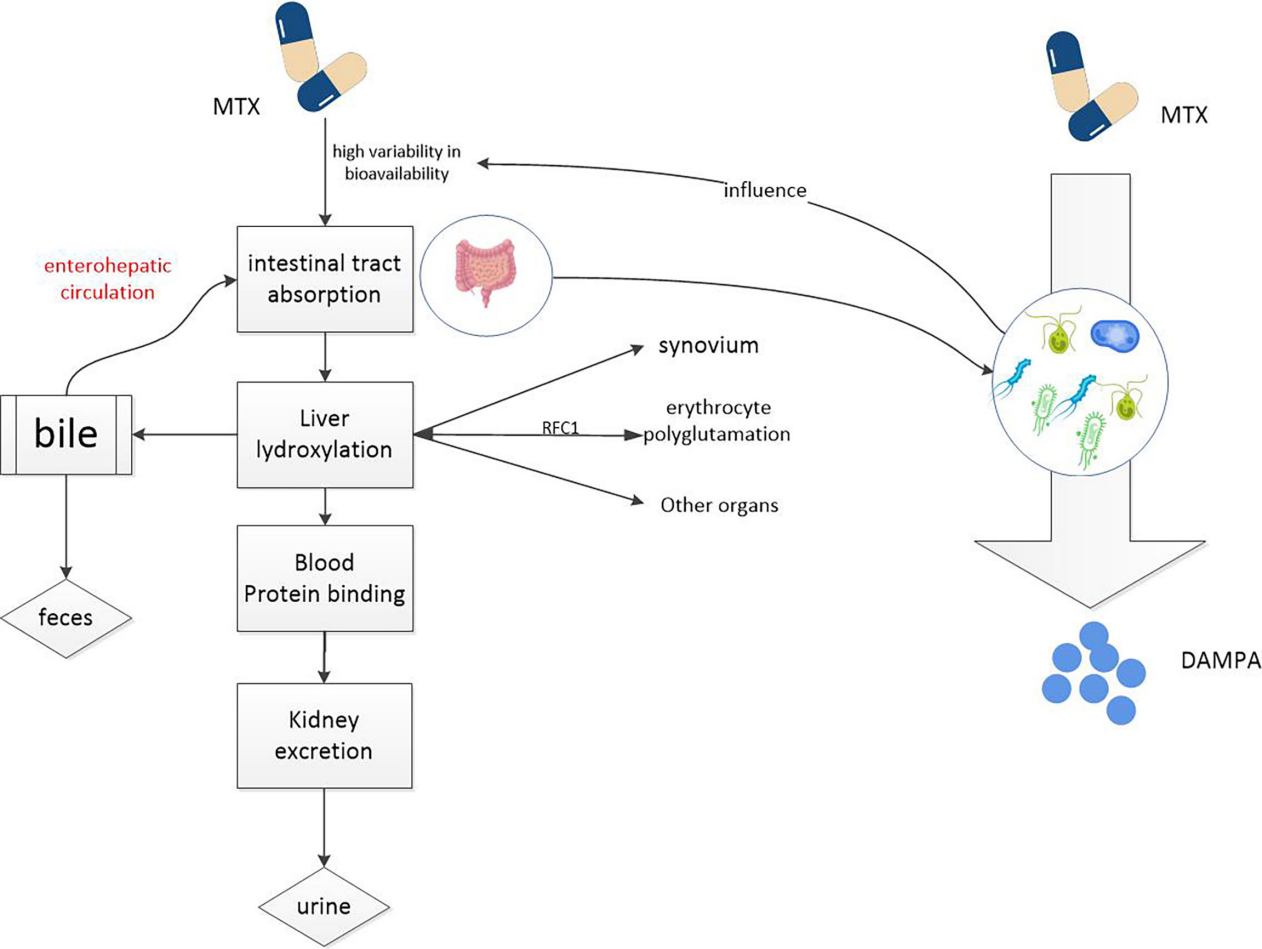
MTX metabolism in the body. MTX enters the circulation from the intestinal tract and accumulates in red and white blood cells, liver cells, and synovial cells, potentially causing toxic reactions. Drugs from the liver can re-enter the intestine through the enterohepatic circulation. It is metabolized into non-toxic DAMPA with the participation of a number of gut microbial enzymes. So gut microbes can reduce the potential toxicity of MTX.

Gut microbiome can directly affect the bioavailability of MTX (19). Glutamate carboxypeptidase 2(CPDG2) is an enzyme found in many gut bacteria. The terminal glutamate residues of MTX and 7-OH-MTX can be cleavaged to generate 2, 4-diamino-N-10-methylpteroic acid (DAMPA) and 7-hydroxy-DAMPA metabolites with the assistance of CPDG2, respectively (41, 46, 47). In addition, other bacterial enzymes such as p-aminobenzoyl-glutamate hydrolase, found in E. coli, have also been shown to catalyze this reaction (48) (see **Figure 2**). The hydrolysis of glutamate by CPDG2 is considered to be a detoxification process. Furthermore, CPDG2 has been approved as a palliative for cancer patients with delayed MTX clearance and acute renal toxicity (24). Several clinical studies have also reported the usefulness of CPDG2 as a rescue drug (49, 50). This is just one example of how the gut microbiome influences MTX metabolism, and there are certainly other, deeper connections between MTX and gut microbes that we need to explore.

### 2.2 Anti-Inflammatory Mechanisms of MTX

There is growing evidence that many of the mechanisms by which MTX inhibits the inflammatory response have been hypothesized. In this section, we focused on exploring the anti-inflammatory mechanism of MTX to guide rational clinical drug use.

MTX and folates. MTX reaction products inhibit dihydrofolate reductase (DHFR) and affect folic acid metabolism, thus restraining purine and pyrimidine synthesis and downstream DNA synthesis (3, 26, 27, 51). The reduction of purine and pyrimidine synthesis suppresses the T lymphocytes, a key cell in the immune response, so MTX has anti-inflammatory effect (26, 52). However, this drug as a folate analogue can cause a decrease in peripheral blood white blood cell count which is still considered to be a toxic reaction in treating inflammatory diseases (3),although the dose of MTX used to treat RA is 100 to 1000 times lower than that used to treat malignancies. It is now common sense that taking folic acid (except on MTX days) can reduce or even prevent the toxic effects of this immunosuppressant in patients (53–55). Given that even supplementation with folic acid during medication can prevent a decrease in peripheral white blood cell count and that MTX can still inhibit progression in patients with rheumatic disease, the anti-inflammatory effect of MTX may not be as necessary to inhibit cell proliferation (3). 

MTX and adenosine. There is growing evidence that MTX causes the release of adenosine, a powerful stimulator of adenosine receptors (including A1a, A2a, A2b and A3) that inhibits nearly all types of inflammatory cells (56). Studies in mice demonstrated that MTX increased the release of adenosine in inflammatory tissues (57, 58). According to a randomized investigational study (59), ingestion of non-selective adenosine receptor antagonist such as caffeine can reduce the body’s response to MTX therapy, which confirmed MTX treatment can increase the body’s really adenosine release. Recently, emerging evidence suggests that the mechanism by which regulatory T cells (Treg, a type of negatively regulated immune cells) reduce the degree of immune response and thus inhibit inflammation may be related to the production and release of adenosine (56, 60). Treg cells produce adenosine through dephosphorylation of ATP, a process mediated by CD39 and CD73 (61–63). If CD39 or CD73 is relatively low on Treg cell level, such as in RA, this process is impeded, resulting in a poor clinical MTX response (64). 

MTX and reactive oxygen species(ROS).Nitric oxide synthase (NOS) is an isoenzyme found in endothelial cells, macrophages, and nerve cells, which normally promotes the production of NO. Tetrahydrobiopterin(BH4) is a cofactor and ligand of endothelial nitric oxide synthease (eNOS).In the absence of BH4, NOS does not catalyze the produce of NO, but instead produces ROS, such as hydrogen peroxide(a damage factor for human tissues and organs), a process known as nitric oxide synthase uncoupling (65–67). It has been proved that MTX can inhibits nitric oxide synthase uncoupling (3). MTX suppresses the reduction of dihydrofolate and dihydrobiopterin(BH2), that is, increases their concentration, which in turn increases the concentration of BH4 [BH2 is restored to BH4 by dihydrofolate reductase (DHFR)], thereby reducing the production of ROS.

MTX and cytokines. MTX alters the cytokine profile, specifically inhibiting the production of pro-inflammatory cytokines (27, 68, 69). It has been shown that T cells isolated and activated from RA patients treated with MTX have a reduced ability to produce inflammatory cytokines such as IFN-γ, IL-4, IL-3, and TNF (70). MTX treatment also reduced the number of TNF-positive CD4 T cells and increased the number of IL-10 CD4 T cells (71). Which is beneficial for RA patients to manage their disease.

**Figure 3:**
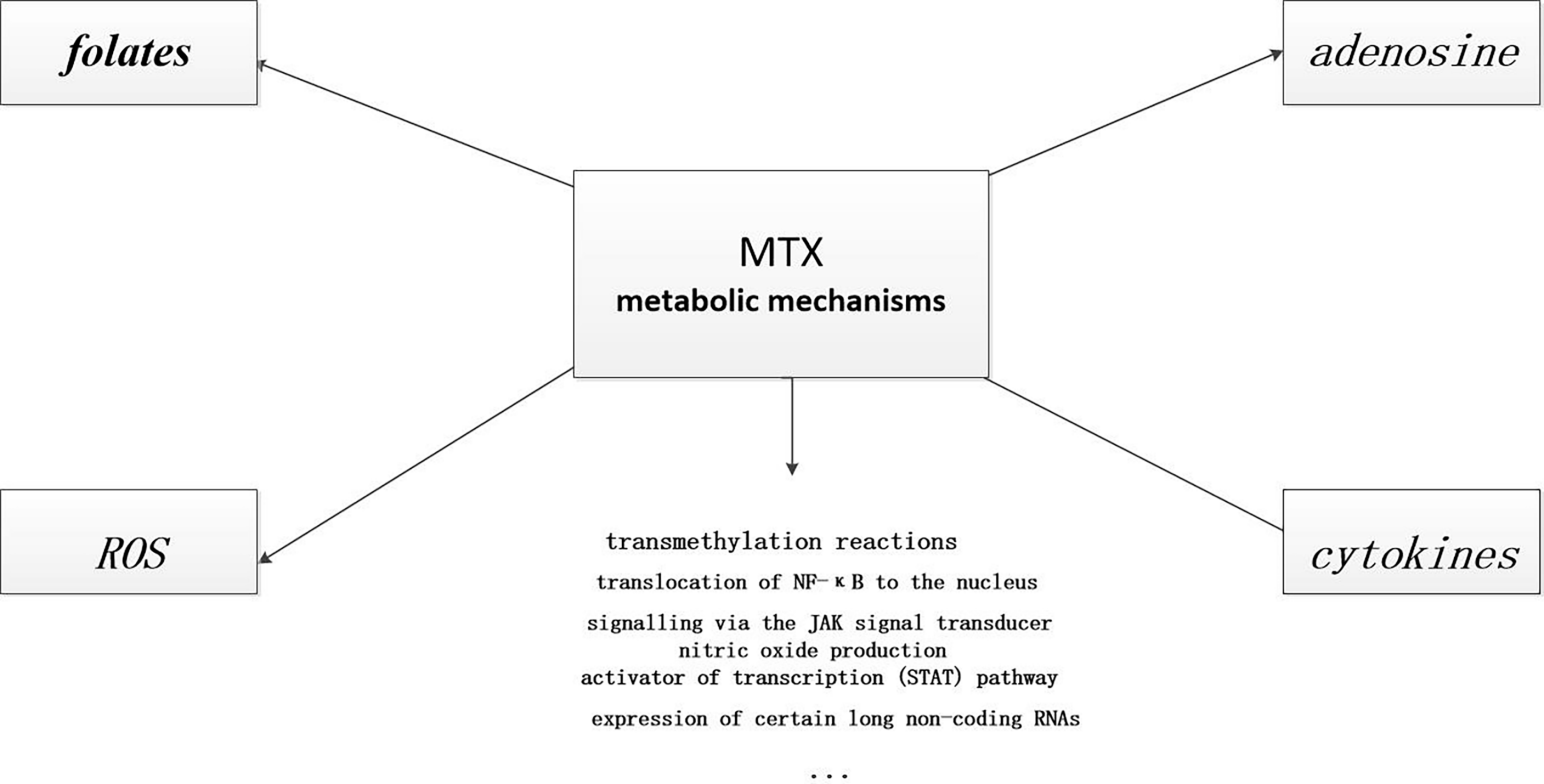
Anti-inflammatory mechanisms of MTX. MTX has many anti-inflammatory mechanisms. Some of these pathways are known, and there are certainly many more that need to be explored.

Other anti-inflammatory mechanisms have also been reported, including inhibition of transmethylation (3), regulation of the expression of some long non-coding RNAs (3), and reduction of chemotaxis and adhesion of inflammatory cells (27) (see **Figure 3**). Considering the important efficacy, wide application and numerous adverse reactions of MTX, further research on the mechanism of action of MTX is to optimize treatment.

### 2.3 Toxic Effects of MTX

In the nearly 40 years of MTX use, an increasing number of toxic effects have been observed, including gastrointestinal, myelotoxicity, cardiotoxicity, nephrotoxicity, and hepatotoxicity. Currently, enterotoxicity is the most common side effect and the main factor limiting its administration, mainly manifested as MTX-induced intestinal mucosal inflammation, which is now increasingly evidenced to be related to gut microbiome (18). It has been suggested in the past that the toxic effects of long-term oral low doses of MTX are inextricably related to the antifolate properties of the drug (3). So it has become common knowledge to take folic acid along with MTX to prevent its toxic effects. Now, the latest international view suggests that the mechanism of folic acid supplementation to reduce the toxic effect may be related to intestinal flora, especially the probiotics such as Bacteroides and Bifidobacterium. And some researchers think that some of the toxic effects of MTX may be caused by MTX-mediated adenosine release. MTX promotes the release of adenosine in the inflammatory sites, which binds to receptors in the brain to suppress the inflammatory response and causes drowsiness (72–74). So many patients feel tired on the day they take MTX and other patients developed rheumatoid nodules (75) and liver fibrosis (76, 77). 

Another serious and unpredictable side effect is pulmonary toxicity, which occurred in 1% of the 500,000 RA patients treated with MTX (78, 79), mainly including interstitial pneumonia and pulmonary fibrosis (80). The mechanisms include genotype mutation and transport inhibition, and are related to the p38 Mitogen-activated protein kinase (MAPK) pathway and interleukin-8 (IL-8) (81). These mechanisms can be used to optimize drug therapy, such as serratiopeptidase (PSTD) combined with fisetin (FST) against MTX-induced pulmonary toxicity (82). One thing to be aware of is hypersensitive pneumonia (HP), a diffuse interstitial lung disease caused by sensitization of susceptible individuals to repeated and prolonged exposure to large amounts of antigen (83). It is often associated with exposure to external allergens (such as poultry dust, mould and tobacco) and medications including cytotoxins (such as MTX) and antiepileptic drugs (such as carbamazepine) (84). Some HP patients may recover, while others develop pulmonary fibrosis, which may be related to the microbiota of the lower respiratory tract (85). For example, Proteobacteria are the predominant in HP, while Firmicutes dominate in pulmonary fibrosis.

However, a recent randomized, double-blind, placebo-controlled trial (86)showed that low doses of MTX reduced the incidence of complications from cardiovascular events in patients with RA and severe cardiovascular risk. Another new and particularly worrisome event is skin cancer (86). Various European biologics registers found a higher incidence of skin cancer in RA patients taking MTX than in the general population (87–89). This has been attributed to long-term immunosuppression, and these new data support this. Long-term oral low-dose MTX, on the one hand, slows disease progression and reduces the risk of subsequent complications such as lymphoma and cardiovascular events, and also increases the incidence of skin cancer.

### 2.4 Therapeutic Implications of MTX

Since the discovery of rheumatoid arthritis in the middle of the last century, MTX is still the gold standard for RA, despite the recent emergence of various drugs. Factors that can predict clinic response and prognosis to the drug are especially important to reduce disease activity and unnecessary potential toxic effects. Lately, a machine learning analysis of whole blood transcriptome data from RA patients (90) holds that gene expression of multiple inflammatory pathways may have predictive value for clinical response to MTX, and that gene expression of anti-I interferon response is the most valuable predictor. However, the results were not satisfactory, and the whole genes containing all the gene expression had higher predictive value than the individual interferon gene alone (3, 90). Meanwhile, several other studies have sought to identify clinical and/or laboratory markers that might predict clinical responses to MTX, but all ended in failure (91–93). Despite high hopes for genetic markers that identify MTX responses, to date, no factor or biomarker has been definitively proven to be predictive. Instead, there is a growing interest in the gut microbiota to influence MTX bioavailability and as a predictor of subsequent clinical responses to MTX in the RA patients. The next section, we will detail the potential interaction between MTX and the gut microbiota.

## 3 Pharmacomicrobiology of MTX in RA

Current treatment guidelines for RA [such as ACR (29) and EULAR (94)] recommend MTX for all patients with early RA, because early and aggressive MTX intervention can slow the onset and progression of RA, reduce disease activity, alleviate clinical symptoms, and even radiographic progression in some RA patients. Therefore, there is an urgent need for in-depth understanding to identify factors and predictors that influence MTX in order to maximize clinical efficacy and mitigate toxic effects, thereby eliminating frustration and reducing the waste of medical expenditures. Currently, a variety of candidate biomarkers have been considered to have potential predictive value for clinical efficacy of MTX, including clinical phenotypes, inflammatory pathway gene expression, host genetics, autoantibodies, and cytokines, etc, involving proteomics, metabolomics, transcriptomics and other omics. Unfortunately, none of them has been able to accurately predict clinical response to MTX (95). 

A growing body of evidence suggests that non-human genetic factors, particularly from the trillions of microorganisms that is the microbiome, may contribute to the development of inflammatory arthritis in genetically susceptible individuals (96–98). Intriguingly, variability in interindividual microbiome composition and metabolic capacity play a unique role in determining the clinical efficacy (and the development associated with adverse events) of certain drugs (99–101). In turn, drugs may also exert their anti-inflammatory effects by altering a patient’s gut microbiome. The study of the interactions between drugs and microorganisms, known as pharmacobiology, has led us to the concept of precision medicine, which is not foreign to most rheumatologists. The long-term and ultimate goal of this discipline is to manipulate the complex host-associated microbiome (both *in vivo* and on the surface of the body), in order to predict clinical responses, improve clinical efficacy, and minimize adverse events (17).

### 3.1 Effect of Gut Microbiome on Clinical Efficacy of MTX in RA

What accounts for variation in clinical outcomes between individuals? The answer is not entirely clear. Moreover, to date, known factors such as host genetics, laboratory or host biomarkers cannot be used to accurately predict clinical response to a drug (95). Furthermore, differences in the metagenomes of microorganisms in the gut of RA patients such as bacterial strains, genes, enzymes, proteins and/or metabolites may at least partially determine their bioavailability and/or subsequent response to MTX. So, there is reason to believe that there is some connection between MTX and the gut microbiota.

#### 3.1.1 Gut Microbiome Partly Determines How RA Patients Respond to MTX

A preliminary study using red blood cell MTX polyglutamate concentration explained 20% of the variation in MTX response, but unfortunately this finding was not consistently reproduced in other cohorts (102–105). Other possible factors including serum or plasma MTX concentration (106, 107), clinical factors such as sex and disease activity (92, 108–110) and circulating CD39+ regulatory T cells (92, 111), and genetic factors such as gene polymorphism (112) as predictors of MTX efficacy all failed. More than a decade ago, the first clinical pharmacological model (113) was proposed to predict the clinical response or not of MTX monotherapy to newly diagnosed RA. This model does not accurately predict the clinical efficacy of RA and cannot be widely used in the population, although it improves on the original genetic-based model by incorporating multiple variables (114–116). 

The fact that so many other factors do not account for individual differences in MTX response raises the possibility that the differences are caused at least in part by individual differences in the metagenome of the gut microbiome. More recently, some research groups have suggested that intestinal microbiota is closely related to immunoregulatory therapy, and that it may have great predictive value in the clinical response of drugs (117–122). Microbial differences found in the gastrointestinal tract of RA patients may partially determine the bioavailability and/or subsequent clinical outcome of MTX. This theory was first demonstrated in rodents. Valerino, D. M., Johns, D. G. et al. (41) found that the intestinal absorption and metabolism of MTX in germ-free and antibiotic-treated mice were reduced compared to wild-type mice, suggesting that the biotransformation of MTX was hindered if the microbiome was not tasted, that is, gut microorganisms is an essential link in the absorption and metabolism of MTX. Moreover, according to a 2013 study (97) found that untreated new RA patients with gut microbiome differences between purine metabolic pathways, including four hydrogen folic acid (and other purines) biosynthesis, adjustable oral absorption and bioavailability of MTX and downstream treatment effect. Another study (123), which differentiated RA patients from healthy controls based on differences in oral microbiota, found that clinical measures such as disease activity and symptoms such as joint pain were strongly associated with changes in the microbiome. This suggests the potential diagnostic and prognostic value of the gut microbiota in RA. However, this study focused on the oral microbiome (which have a lesser similarity to gut microbiota) as a predictor, focusing primarily on confirmed patients with a long-term RA, who had a significantly different microbiome from the new-onset (NORA) subjects.

A 2020 study (124) delved into the relationship between gut microbes and oral MTX response in NORA. They found that the gut microbial community structure before MTX administration, made the difference between clinical response or not, which is that MTX-responders (MTX-R) has significantly lower microbial diversity compared with non-responder (MTX-NR). At the phylum level, the ratio of Firmicutes/Bacteroidetes in MTX-NR is high. At the same time, MTX-rich bacteria in NR samples are: the Euryarchaeota phylum,unclassified Clostridiales/Clostridiales Incertae Sedis XIII (family) and Escherichia/Shigella. By contrast, in MTX-R, Prevotella and Bacteroides genera are significantly more abundant. The team then used shotgun sequencing to define differences in metagenome and gene abundance of gut bacteria between the two groups and found 6,356 KEGG Orthologs (KOs). 7 microbial modules (mainly involves metabolic and biosynthetic potential) and 462 KOs were identified for the separation of MTX-NR and MTX-R. For instance, many pathways including MAPK signaling pathway, fatty acid degradation and DNA replication were significantly increased in MTX-NR. The 2013 study (97)also distinguished MTX responders from non-responders in terms of microbial metabolic pathways. To sum up, we conclude gut bacteria metagenome including microbial or bacterial function may be more likely to be related to clinical responses than microbial community structure.

Taken together, NORA patients who responded well to MTX had a different gut microbiome than MTX-NR, which allows us to predict the clinical absence of MTX by using pretreated microbiota.

#### 3.1.2 Pharmacobiological Methods Can Be Used to Predict the Clinical Efficacy of MTX in RA Patients

Research groups studying human autoimmune diseases use pharmacobiological methods to analyze the gut microbiome and/or its gene coding function as predictors of biotherapy response (17). The use of gut microbiome to predict the clinical respond of MTX in newly diagnosed RA patients was included. These research groups came to two conclusions:”gut metagenome at baseline could differentiate MTX responders from non- responders” and another is that “*ex vivo* incubation with MTX of samples from patients with treatment-​naive, new-​onset RA correlated with the magnitude of future clinical response”.And accordingly, a small idea that a new and potentially valuable tool (metagenome-based classifiers) may be available for the decision-making of newly emerging RA was put forward. That’ s exactly what the 2020 study has found.

Data from this 2020 study (124) demonstrate that gut metagenomics at baseline can predict MTX clinical response or not and provide a predictive model for it, as well as a possible plausible mechanism for the relationship between gut microbiome and clinical response to MTX prior to treatment. As described earlier, there were significant differences in gut metagenomes between MTX-NR and MTX-R patients prior to the start of treatment. Therefore, machine learning based on gut microbiome metagenomes can robustly predict MTX responses. The research group built a random forest model to predict whether MTX would respond or not. At the same time, they proved that a model based on microbiome characteristics, rather than clinical pharmacological characteristics, can determine response to MTX. However, the potential clinical application of this model is limited to drug-naive and RA patients with direct exposure to MTX. Although this model has many deficiencies, it has contributed greatly to the prediction of clinical response of MTX by intestinal metagenomics at baseline compared to the previous model (113). 

Intestinal metagenomes with significant differences before treatment initiation can predict clinical response to MTX. And why is this? Let’s put forward a hypothesis that the gut metagenomes are associated with known MTX metabolic pathways. And This hypothesis has been confirmed. As what mentioned before, a 2013 study (97) found that untreated new RA patients with gut microbiome differences between purine metabolic pathways. The MTX-NR microbiome showed the increased genes abundance was mainly concentrated in the genes encoding for purine nucleoside phosphorylase and adenine deaminase, which increased hypoxanthine and reduced the bioavailability and cytotoxicity of MTX (125, 126). In contrast, the gene-coding enzymes (i.e. hisH and hisA) associated with the accumulation of 5-aminimidazole-4-carboxylate ribonucleoside (AICAR) were relatively reduced in MTX-NR (127). These two enzymes can regulate many immune-mediated effects of MTX by regulating the amount of AICAR. These two examples further highlight the importance of characterization of gut metagenomic, including enzymes or proteins, for the development of drug metabolic functions. Unfortunately, given that the gut metagenome contains a collection of all the genomes in the microbiome, it is difficult to determine the efficacy of MTX at the individual level and in clinical practice, although it can distinguish MTX significantly at the group level. If cost is taken into account, the actual situation becomes more complicated.

Then the group (124) used two independent metabolomic platforms to confirme that the differences in MTX clinical responses were directly mediated by NORA gut microbiome *via* affecting its metabolism. This provides for the first time a possible mechanism for the association between gut microbiome and drug response, that is to say, “gut bacteria derived from MTX non-responders differentially deplete MTX *ex vivo* and remaining drug levels correlate with decreased clinical response”. First, the ability of the fecal microbiota to metabolize MTX *in vivo* is highly variable between individuals. Then, fecal microbiome in the MTX-NR group could rapidly reduce the concentration of MTX, while the concentration of MTX in the fecal supernatant of the MTX-R group remained unchanged. Last, the MTX elimination rate in each individual was significantly inversely correlated with future observed clinical responses. MTX levels measured in the supernatant related to future clinical responses when incubated with fecal samples. Therefore, patients’ gut microbiome may be a predictor of clinical response to MTX.

**Figure 4:**
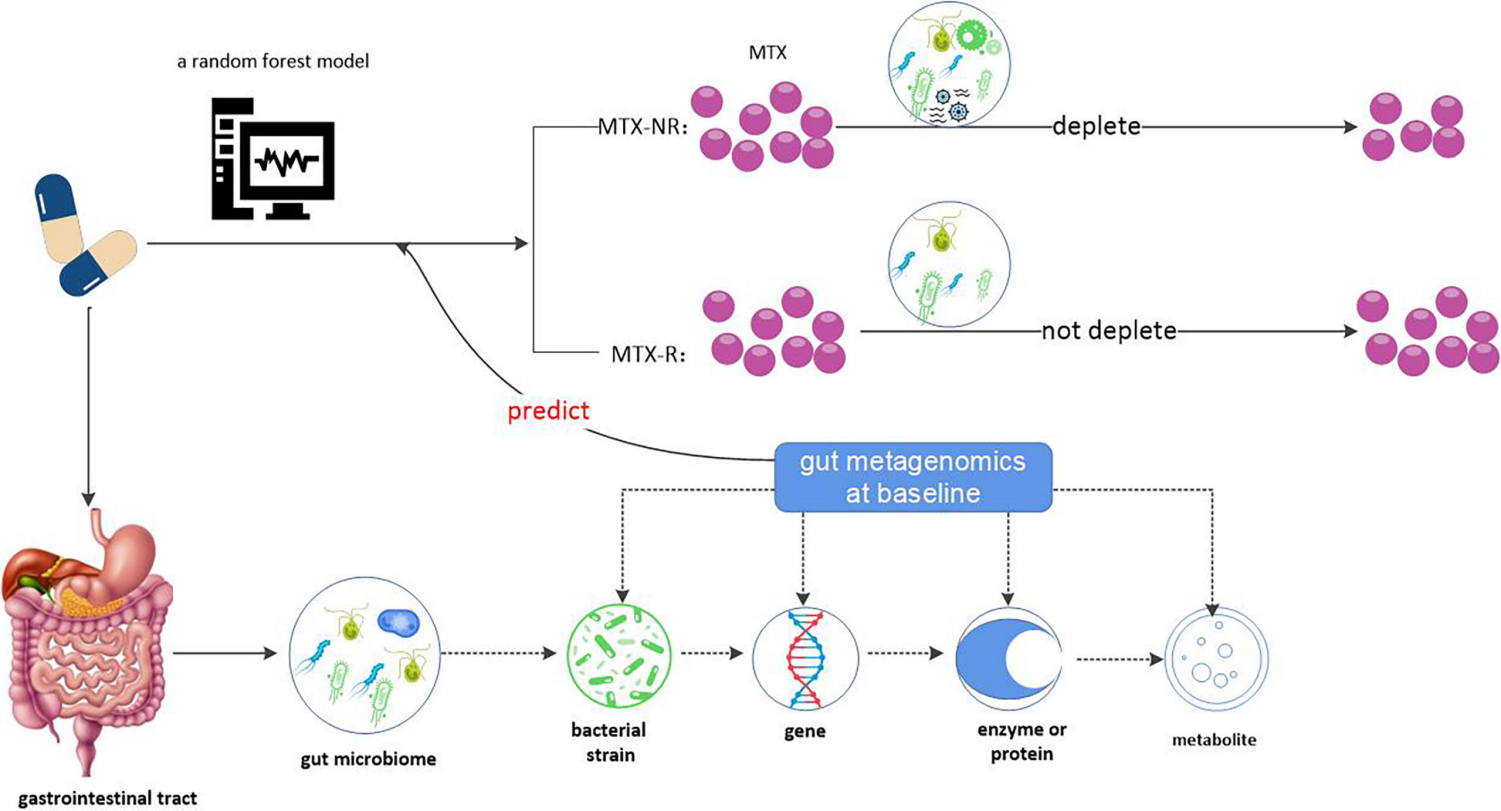
Gut microbiome may be a predictor of clinical response to MTX. Gut metagenome at baseline could differentiate MTX responders from non- responders and MTX-R has significantly lower microbial diversity compared with MTX-NR. According to this, a random forest model based on microbiome characteristics had be built. Why does the gut metagenome determine clinical response to MTX? One possible reason is that gut bacteria affect MTX metabolism. Gut bacteria derived from MTX non-responders differentially deplete MTX *ex vivo* and remaining drug levels correlate with decreased clinical response. Therefore, patients’ gut microbiome may be a predictor of clinical response to MTX.

Taken together, we obtained the conclusion that gut microbiome (more accurately, gut microbiome metagenome) may serve as a predictor of clinical MTX response and the possible mechanism by which gut microbiome acts on MTX, where patients with more abundant gut bacteria were able to efficiently metabolize and/or consume MTX, and were associated with worsening clinical outcomes (see **Figure 4**). 

### 3.2 Influence of Gut Microbiome or Its Metabolites on MTX Gastrointestinal Toxicity

Long-term oral administration of MTX may cause many adverse reactions, including intestinal toxicity, cardiac toxicity, bone marrow toxicity, renal toxicity and pulmonary toxicity, which greatly limits the clinical application of MTX (128, 129). Among them, intestinal toxicity is the most common side effect (18). The main symptoms include nausea, vomiting, abdominal pain, bloating, and diarrhea, leading to malabsorption, weight loss, and eventually discontinuation of chemotherapy (25, 128, 130). It is possible that MTX exerts toxic effects on normal intestinal cells by disrupting metabolic pathways such as anti-inflammatory, anti-oxidant and apoptosis (131). Another possible mechanism is that changes in gut microorganisms or their metabolites cause polarization of macrophages, leading to the release of inflammatory factors such as interleukin-12 (IL-12) and tumor necrosis factor (TNF), and ultimately aggravate gut mucosal injury. However, it is a complex process for MTX to cause gastrointestinal damage, which is not entirely clear so far. Therefore, there are no satisfactory therapeutic interventions to prevent or treat this adverse reaction.

The homeostasis of the intestinal environment is critical to the integrity and function of the entire gastrointestinal tract (132). The intestinal microbiota plays an important role in the development and progression of mucositis, which is the central manifestation of intestinal damage (133). Changes in the composition, structure and metabolites of the gut microbiome may affect the metabolism of MTX, thus affecting the status of gut mucosa. However, these complex dynamic processes need further investigation.

Some bacteria (18) or metabolites (134, 135) including amino acids, polyphenols or vitamins have been reported to influence MTX-induced intestinal mucosal injury. One possible explanation is that chronic exposure to MTX may induce community and functionality changes in the intestinal, and induce disruption of downstream CPDG2 activity, which may delay the detoxification of DAMPA by MTX, leading to increased gastrointestinal toxicity (19). However, each strain or metabolite administered individually has a limited range of effects on gastrointestinal toxicity. Therefore, it is feasible to reduce the gastrointestinal toxicity of MTX by establishing a combination therapy with candidate bacteria and metabolites (136). It has also been reported that changes in diet cause changes in the microenvironment of the gut microbiota, which ultimately aggravates MTX-induced intestinal inflammation (136). The proteins or lipids in high-fat high-sucrose diet(HFHSD) alter the intestinal environment and affect the pathogenesis of MTX-induced intestinal mucosal injury.

At present, treatment with leucovorin (LV) is the most common method to reduce the toxic effects of MTX. Previous studies (137, 138) have confirmed that the decrease in the severity of intestinal inflammation after the combined administration of MTX and LV is related to the diversity, richness and species composition of gut microbial community. But not all species of gut bacteria are affected to the same degree. A recent study (139) has shown that LV can improve MTX intestinal toxicity, possibly *via* increasing the composition of beneficial bacteria such as Bifidobacterium and reversing the imbalance of intestinal microbiota caused by MTX. A 2018 study (140) also demonstrated the protective effect of Bacillus fragilis on MTX-induced inflammation. Of course, there is still a need to use metagenomic sequencing to find other beneficial species in further study.

To sum up, both diet and LV reduce gastrointestinal toxicity by affecting the gut microbiome. And it has previously been reported that artificially increased probiotics intake or the use of fecal microbiota transplantation (FMT) ameliorates drug-induced intestinal damage (140). Therefore, we speculate that probiotics and/or FMT may be a safer and more effective method to reduce MTX-induced gut inflammation. However, a recent experiment seems to disagree. This is a double-blind, parallel-group, placebo-controlled, superiority trial (141), showing that FMT from selected donors can worsen psoriatic arthritis (PsA) symptoms. The failure rate (i.e., patients requiring intensive treatment) was significantly higher in the FMT group than in the control group. Therefore, other mechanisms, such as the degree and persistence of donor microbiota implantation, and some external factors related to FMT, such as aerobic and anaerobic environment (141), need to be further attended in the future.

### 3.3 Effects of MTX on Gut Microbiome in RA Patients

Gut microbiome can be used as a predictor of clinical response to MTX, so, conversely, does MTX affect gut microbiome? The answer is of course yes. According a using 16 s - seq study (142),with the passage of time, the routine oral dose MTX will not lead to a gut microbial ecology of persistent disturbances. But many subsequent experiments have demonstrated that MTX can also affect the community structure of intestinal microorganisms, regulating their diversity and function (17). It has recently been reported that MTX inhibits the growth of 30% of 40 representative intestinal bacterial strains and 84% of 43 bacterial isolates (143), covering 43% of the human gut microbiota in their combined relative abundance (17). 

MTX in both *in vitro* and the biological experiments can affect the humanized mice in dose dependent manner the composition of gut microbiome (19). Low doses of MTX increased the species richness and diversity of the microbiota (123, 144), including increasing relative abundance of Firmicutes and decreasing relative abundance of Bacteroidetes, which reverse the perturbations of the microbiota normally associated with RA (17). However, high doses of MTX significantly reduced intestinal bacterial diversity (145). Most of the changes were due to a relative decrease in anaerobic bacteria of Firmicutes, accompanied by a relative increase in Bacteroidetes. High doses of MTX may alter the function or abundance of bacterial enzymes involved in MTX hydrolysis, such as CPDG2, which we mentioned earlier (145). Other studies have shown that MTX exerts an anti-inflammatory effect *via* interacting with off-target bacterial enzymes such as DHFR in E. coli or Lactobacillus Casey (146). And low-dose MTX-induced changes in the gut microbiome reduce host immune activation (147).  Specifically, the microflora changes lead to the reduction of multiple host cell populations, such as activated T cells, B cells, myeloid cells, etc. At the same time, reduced stimulation of immune system activation and decreased tolerance to Treg cell induction suggested that MTX-induced changes in the microbial community reduced its possible inflammatory potential.

A recent study shows that gut microbiota is closely correlated with P – glycoprotein (P-gp, a glycoprotein associated with MTX efficacy) (148). For example, Lachnoclostridium is positively correlated with P-gp, while Turicibacter is negatively correlated with P-gp. As mentioned above, MTX has a significant impact on the gut microbiome of RA patients according to its dose, and combined with the relationship between gut microbiome and P-gp, it is reasonable to speculate that oral MTX affects the therapeutic effect of the drug itself by affecting the gut microbiome. Unfortunately, so far, this idea has only existed in theory and has not been confirmed experimentally.

**Figure 5:**
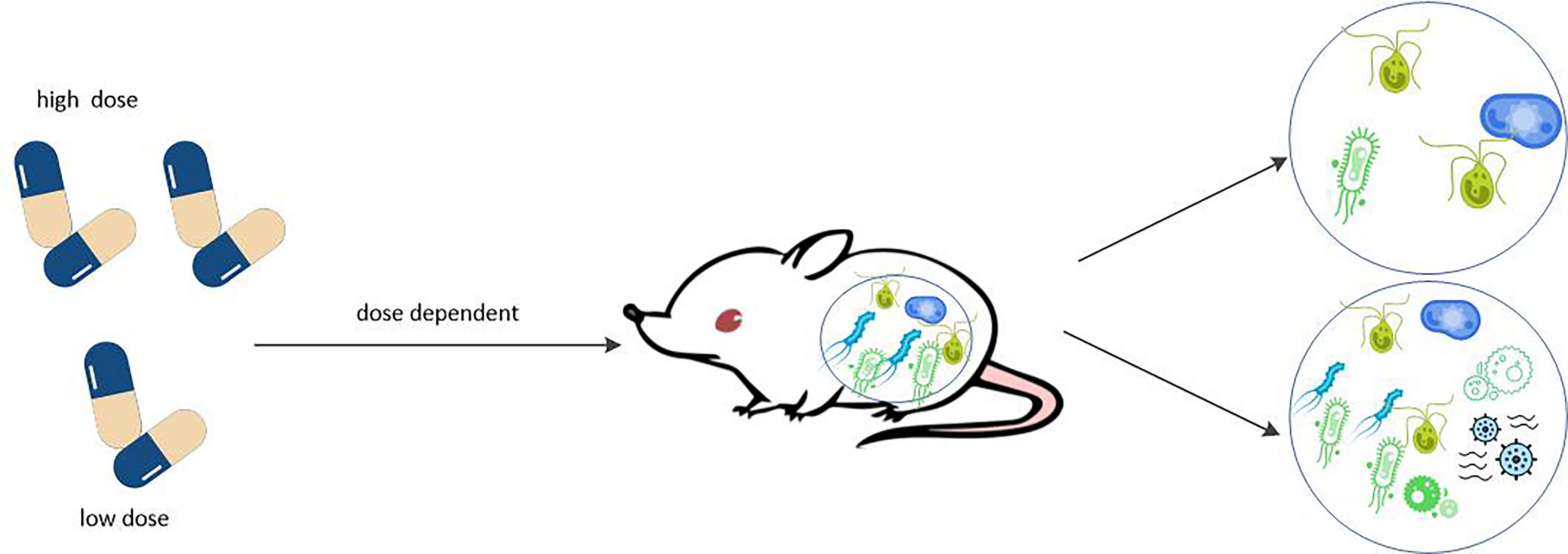
MTX affects the composition of gut microbiome in dose dependent manner. MTX can affect the humanized mice in dose dependent manner the composition of gut microbiome. Low doses of MTX increased the species richness and diversity of the microbiota, which reverse the perturbations of the microbiota normally associated with RA. However, high doses of MTX significantly reduced intestinal bacterial diversity.

The gut metagenome of RA patients before treatment determines whether or not they respond to MTX, and that *in vitro* incubation is correlated with the clinical response intensity. In turn, different doses of MTX altered the species richness and diversity of intestinal bacteria (see **Figure 5**). In other words, there is a dose-dependent bidirectional interaction between MTX and gut microbiota. This interaction between drugs and microbes is known as pharmacobiology, a term not foreign to rheumatologists.

### 3.4 Applications for Precision Medicine of Pharmacobiology in RA

The study of the interaction between drugs and microbes is known as pharmacobiology, leading to the concept of precision medicine, which is particularly important in the field of rheumatic diseases. The research focuses on the genes and enzymes encoded by the entire microbial community, including gut microbiome, as well as the relationship between microorganisms and pharmacokinetics, which ultimately modulates host immune response in order to treat diseases (121). The understanding of pharmacomicrobiology has led to a new level of understanding of the clinical efficacy and adverse reactions of drugs (17). The integration of precision medicine strategies based on the characteristics of gut microbes can help guide the appropriate use of many drugs, including MTX, which often present known and unpredictable individual differences. Interindividual microbial metagenomes such as bacterial species, genes, enzymes, proteins, and/or metabolites affect drug uptake, metabolism, transport, and/or excretion, which allows clinicians to pretreat patients’ gut microbiome and take the best treatment to get the best treatment results (121). At the same time, this information can guide decisions to try to achieve an ideal microbial composition or gene to improve drug bioavailability and improve symptoms. As mentioned above, the use of baseline gut microbiome metagenomes has important predictive value for clinical response to MTX administration, which has been used for inflammatory arthritis. A related example is the FLORA study (149). This is a randomized, placebo-controlled trial of FMT in patients with active PsA who have no response to MTX.

Although FMT has made some progress, other less cumbersome microbial modulators are being tested, including adjuvant probiotics and probiotics, which have shown positive results (137). New technologies such as organ-on-a-chip (150, 151) and bacterial culture (152) help to understand the mechanisms of pharmacomicrobiology *via* attempting to mimic the gut environment and reproduce the physiological interactions of host microbes. Then, the drugs to be studied are cultivated in these systems to explore the interaction between drugs and microorganisms, including the effects of drugs on bacterial metabolism and reproduction, and the effects and mechanisms of bacteria on drug biotransformation. These pharmacobiological studies will provide a new idea for the treatment of rheumatoid arthritis and even inflammatory arthritis.

## 4 Conclusions and Perspectives

Although several efficacious therapies have recently been developed, MTX has always been the basic and first-line drug for the treatment of RA. Multiple mechanisms may contribute to the anti-inflammatory effects of MTX including inhibition of purine and pyrimidine synthesis, promotion of adenosine and some cytokines release, inhibition of ROS, etc., as well as transmethylation reaction, activator (STAT) pathway and expression of some long non-coding RNAs, which are not described in detail in this paper (3, 27). However, there is still a high proportion of RA patients who do not respond or have major toxic effects to MTX. We speculate that this inter-individual difference is at least partly related to the gut microbes of patients, and more and more trials have proved this speculation to be correct. In other words, gut microbes are associated with MTX resistance or poor response. Gut metagenome at baseline could differentiate MTX responders from non- responders and *ex vivo* incubation with MTX of samples from patients with treatment-​naive, new-​onset RA correlated with the magnitude of future clinical response. In turn, MTX affected the composition of intestinal microbes in humanized mice in a dose-dependent manner, inhibiting the growth of certain intestinal bacteria. Although comparison of MTX consumption rate and response probability by metagenome-based models did not find a significant correlation, this may indicate that most gene orthologs included in the model do not appear to play an important role in MTX depletion. A possible explanation is that these gene orthologs may affect the MTX response, but independently of drug metabolism. Future research needs to determine whether the microbiome can directly metabolize MTX in the body, jump-start the immune system to boost the response, or both. The rigorous integration of clinical factors, host genomics, and pharmacobiology will lay the foundation for major advances in rheumatic precision medical. More research is needed on the interactions between drugs and gut bacteria, known as pharmacobiology, which will be a research hotspot now and even in the future in the field of rheumatic diseases including RA.

## Author Contributions

HY drafted the manuscript, prepared illustrations and discussed the content with the other authors. CW conceived the topic and revised the manuscript. RS intellectually revised the manuscript. HX, CG and XL also critically revised the manuscript for intellectual content. All the authors approved the manuscript for publication.

## Funding

This work was supported by National Natural Science Foundation of China (No. 81971543, No. 81471618); Key Research and Development (R&D) Projects of Shanxi Province (201803D31119).

## Conflict of Interest

The authors declare that the research was conducted in the absence of any commercial or financial relationships that could be construed as a potential conflict of interest.

## Publisher’s Note

All claims expressed in this article are solely those of the authors and do not necessarily represent those of their affiliated organizations, or those of the publisher, the editors and the reviewers. Any product that may be evaluated in this article, or claim that may be made by its manufacturer, is not guaranteed or endorsed by the publisher.

## References

[1] Weyand CM Goronzy JJ. The Immunology of Rheumatoid Arthritis. Nat Immunol (2021) 22 10-8(1):10–8. doi: 10.1038/s41590-020-00816-x PMC8557973 33257900

[2] Buch MH Eyre S McGonagle D. Persistent Inflammatory and Non-Inflammatory Mechanisms in Refractory Rheumatoid Arthritis. Nat Rev Rheumatol (2021) 17(1):17–33. doi: 10.1038/s41584-020-00541-7 33293696

[3] Cronstein BN Aune TM. Methotrexate and Its Mechanisms of Action in Inflammatory Arthritis. Nat Rev Rheumatol (2020) 16(3):145–54. doi: 10.1038/s41584-020-0373-9 32066940

[4] Weinblatt ME. Methotrexate: Who Would Have Predicted Its Importance in Rheumatoid Arthritis? Arthritis Res Ther (2018) 20(1):103. doi: 10.1186/s13075-018-1599-7 29848356 PMC5977479

[5] Emery P Breedveld FC Hall S Durez P Chang DJ Robertson D. Comparison of Methotrexate Monotherapy With a Combination of Methotrexate and Etanercept in Active, Early, Moderate to Severe Rheumatoid Arthritis (COMET): A Randomised, Double-Blind, Parallel Treatment Trial. Lancet (2008) 372(9636):375–82. doi: 10.1016/s0140-6736(08)61000-4 18635256

[6] Detert J Bastian H Listing J Weiß A Wassenberg S Liebhaber A. Induction Therapy With Adalimumab Plus Methotrexate for 24 Weeks Followed by Methotrexate Monotherapy Up to Week 48 Versus Methotrexate Therapy Alone for DMARD-Naive Patients With Early Rheumatoid Arthritis: HIT HARD, an Investigator-Initiated Study. Ann Rheum Dis (2013) 72(6):844–50. doi: 10.1136/annrheumdis-2012-201612 22739990

[7] Qin J Li R Raes J Arumugam M Burgdorf KS Manichanh C. A Human Gut Microbial Gene Catalogue Established by Metagenomic Sequencing. Nature (2010) 464(7285):59–65. doi: 10.1038/nature08821 20203603 PMC3779803

[8] Lorenzo D GianVincenzo Z Carlo Luca R Karan G Jorge V Roberto M. Oral-Gut Microbiota and Arthritis: Is There an Evidence-Based Axis? J Clin Med (2019) 8(10):1753. doi: 10.3390/jcm8101753 PMC6832398 31652577

[9] Ivanov II Honda K. Intestinal Commensal Microbes as Immune Modulators. Cell Host Microbe (2012) 12(4):496–508. doi: 10.1016/j.chom.2012.09.009 23084918 PMC3516493

[10] Sommer F Bäckhed F. The Gut Microbiota–Masters of Host Development and Physiology. Nat Rev Microbiol (2013) 11(4):227–38. doi: 10.1038/nrmicro2974 23435359

[11] Bäumler AJ Sperandio V. Interactions Between the Microbiota and Pathogenic Bacteria in the Gut. Nature (2016) 535(7610):85–93. doi: 10.1038/nature18849 27383983 PMC5114849

[12] Kamada N Seo SU Chen GY Núñez G. Role of the Gut Microbiota in Immunity and Inflammatory Disease. Nat Rev Immunol (2013) 13(5):321–35. doi: 10.1038/nri3430 23618829

[13] Alexander JL Wilson ID Teare J Marchesi JR Nicholson JK Kinross JM. Gut Microbiota Modulation of Chemotherapy Efficacy and Toxicity. Nat Rev Gastroenterol Hepatol (2017) 14(6):356–65. doi: 10.1038/nrgastro.2017.20 28270698

[14] Koh A De Vadder F Kovatcheva-Datchary P Bäckhed F. From Dietary Fiber to Host Physiology: Short-Chain Fatty Acids as Key Bacterial Metabolites. Cell (2016) 165(6):1332–45. doi: 10.1016/j.cell.2016.05.041 27259147

[15] Li H He J Jia W. The Influence of Gut Microbiota on Drug Metabolism and Toxicity. Expert Opin Drug Metab Toxicol (2016) 12(1):31–40. doi: 10.1517/17425255.2016.1121234 26569070 PMC5683181

[16] Wang J Shao L Rao T Zhang W Huang WH. Chemo-Preventive Potential of Falcarindiol-Enriched Fraction From Oplopanax Elatus on Colorectal Cancer Interfered by Human Gut Microbiota. Am J Chin Med (2019) 47(6):1381–404. doi: 10.1142/s0192415x1950071x 31488036

[17] Scher JU Nayak RR Ubeda C Turnbaugh PJ Abramson SB. Pharmacomicrobiomics in Inflammatory Arthritis: Gut Microbiome as Modulator of Therapeutic Response. Nat Rev Rheumatol (2020) 16(5):282–92. doi: 10.1038/s41584-020-0395-3 PMC11221369 32157196

[18] Zhou B Xia X Wang P Chen S Yu C Huang R. Induction and Amelioration of Methotrexate-Induced Gastrointestinal Toxicity Are Related to Immune Response and Gut Microbiota. EBioMedicine (2018) 33:122–33. doi: 10.1016/j.ebiom.2018.06.029 PMC6085585 30049384

[19] Letertre MPM Munjoma N Wolfer K Pechlivanis A McDonald JAK Hardwick RN. A Two-Way Interaction Between Methotrexate and the Gut Microbiota of Male Sprague-Dawley Rats. J Proteome Res (2020) 19(8):3326–39. doi: 10.1021/acs.jproteome.0c00230 PMC7426014 32544340

[20] Saad R Rizkallah MR Aziz RK. Gut Pharmacomicrobiomics: The Tip of an Iceberg of Complex Interactions Between Drugs and Gut-Associated Microbes. Gut Pathog (2012) 4(1):16. doi: 10.1186/1757-4749-4-16 23194438 PMC3529681

[21] Doestzada M Vila AV Zhernakova A Koonen DPY Weersma RK Touw DJ. Pharmacomicrobiomics: A Novel Route Towards Personalized Medicine? Protein Cell (2018) 9(5):432–45. doi: 10.1007/s13238-018-0547-2 PMC5960471 29705929

[22] Visser K van der Heijde D. Optimal Dosage and Route of Administration of Methotrexate in Rheumatoid Arthritis: A Systematic Review of the Literature. Ann Rheum Dis (2009) 68(7):1094–9. doi: 10.1136/ard.2008.092668 PMC2689521 19033290

[23] Nathan PC Whitcomb T Wolters PL Steinberg SM Balis FM Brouwers P. Very High-Dose Methotrexate (33.6 G/M(2)) as Central Nervous System Preventive Therapy for Childhood Acute Lymphoblastic Leukemia: Results of National Cancer Institute/Children's Cancer Group Trials CCG-191p, CCG-134P and CCG-144p. Leuk Lymphoma (2006) 47(12):2488–504. doi: 10.1080/10428190600942769 17169794

[24] Rattu MA Shah N Lee JM Pham AQ Marzella N. Glucarpidase (Voraxaze), a Carboxypeptidase Enzyme for Methotrexate Toxicity. P T (2013) 38(12):732–44. PMC3875266 24391395

[25] Howard SC McCormick J Pui CH Buddington RK Harvey RD. Preventing and Managing Toxicities of High-Dose Methotrexate. Oncologist (2016) 21(12):1471–82. doi: 10.1634/theoncologist.2015-0164 PMC5153332 27496039

[26] Maksimovic V Pavlovic-Popovic Z Vukmirovic S Cvejic J Mooranian A Al-Salami H. Molecular Mechanism of Action and Pharmacokinetic Properties of Methotrexate. Mol Biol Rep (2020) 47(6):4699–708. doi: 10.1007/s11033-020-05481-9 32415503

[27] Friedman B Cronstein B. Methotrexate Mechanism in Treatment of Rheumatoid Arthritis. Joint Bone Spine (2019) 86(3):301–7. doi: 10.1016/j.jbspin.2018.07.004 PMC6360124 30081197

[28] Lee J Pelkey R Gubitosa J Henrick MF Ganz ML. Comparing Healthcare Costs Associated With Oral and Subcutaneous Methotrexate or Biologic Therapy for Rheumatoid Arthritis in the United States. Am Health Drug Benefits (2017) 10(1):42–9. PMC5394544 28465768

[29] Singh JA Saag KG Bridges SL Jr. Akl EA Bannuru RR Sullivan MC. 2015 American College of Rheumatology Guideline for the Treatment of Rheumatoid Arthritis. Arthritis Care Res (Hoboken) (2016) 68(1):1–25. doi: 10.1002/acr.22783 26545825

[30] Smolen JS Landewé R Bijlsma J Burmester G Chatzidionysiou K Dougados M. EULAR Recommendations for the Management of Rheumatoid Arthritis With Synthetic and Biological Disease-Modifying Antirheumatic Drugs: 2016 Update. Ann Rheum Dis (2017) 76(6):960–77. doi: 10.1136/annrheumdis-2016-210715 28264816

[31] Morsy MA Ibrahim SA Amin EF Kamel MY Rifaai RA Hassan MK. Curcumin Ameliorates Methotrexate-Induced Nephrotoxicity in Rats. Adv Pharmacol Sci (2013) 2013387071:1–7. doi: 10.1155/2013/387071 PMC3870078 24381587

[32] Widemann BC Balis FM Kempf-Bielack B Bielack S Pratt CB Ferrari S. High-Dose Methotrexate-Induced Nephrotoxicity in Patients With Osteosarcoma. Cancer (2004) 100(10):2222–32. doi: 10.1002/cncr.20255 15139068

[33] Perez-Verdia A Angulo F Hardwicke FL Nugent KM. Acute Cardiac Toxicity Associated With High-Dose Intravenous Methotrexate Therapy: Case Report and Review of the Literature. Pharmacotherapy (2005) 25(9):1271–6. doi: 10.1592/phco.2005.25.9.1271 16164401

[34] Schiff MH Sadowski P. Oral to Subcutaneous Methotrexate Dose-Conversion Strategy in the Treatment of Rheumatoid Arthritis. Rheumatol Int (2017) 37(2):213–8. doi: 10.1007/s00296-016-3621-1 28012023

[35] van Roon EN van de Laar MA. Methotrexate Bioavailability. Clin Exp Rheumatol (2010) 28(5 Suppl 61):S27–32. 21044430

[36] Li CKH Baker K Jones T Coulson E Roberts A Birrell F. Safety and Tolerability of Subcutaneous Methotrexate in Routine Clinical Practice. Arthritis Care Res (Hoboken) (2021) 73(9):1306–11. doi: 10.1002/acr.24334 32475009

[37] Goodman SM Cronstein BN Bykerk VP. Outcomes Related to Methotrexate Dose and Route of Administration in Patients With Rheumatoid Arthritis: A Systematic Literature Review. Clin Exp Rheumatol (2015) 33(2):272–8. PMC4406815 25536122

[38] Genestier L Paillot R Quemeneur L Izeradjene K Revillard JP. Mechanisms of Action of Methotrexate. Immunopharmacology (2000) 47(2-3):247–57. doi: 10.1016/s0162-3109(00)00189-2 10878292

[39] Goldman ID Matherly LH. The Cellular Pharmacology of Methotrexate. Pharmacol Ther (1985) 28(1):77–102. doi: 10.1016/0163-7258(85)90083-x 2414788

[40] Zaharko DS Bruckner H Oliverio VT. Antibiotics Alter Methotrexate Metabolism and Excretion. Science (1969) 166(3907):887–8. doi: 10.1126/science.166.3907.887 5345205

[41] Valerino DM Johns DG Zaharko DS Oliverio VT. Studies of the Metabolism of Methotrexate by Intestinal Flora. I. Identification and Study of Biological Properties of the Metabolite 4-Amino-4-Deoxy-N 10 -Methylpteroic Acid. Biochem Pharmacol (1972) 21(6):821–31. doi: 10.1016/0006-2952(72)90125-6 5014749

[42] Griffin D Said HM. The Enterohepatic Circulation of Methotrexate *In Vivo*: Inhibition by Bile Salt. Cancer Chemother Pharmacol (1987) 19(1):40–1. doi: 10.1007/bf00296253 3815724

[43] Trifunović J Borčić V Vukmirović S Vasović V Mikov M. Bile Acids and Their Oxo Derivatives: Environmentally Safe Materials for Drug Design and Delivery. Drug Chem Toxicol (2017) 40(4):397–405. doi: 10.1080/01480545.2016.1244680 27780364

[44] Vallon V Osswald H. Adenosine Receptors and the Kidney. Handb Exp Pharmacol (2009) 193(chapter 15):443-70. doi: 10.1007/978-3-540-89615-9_15 PMC6027627 19639291

[45] Nuernberg B Koehnke R Solsky M Hoffman J. Furst DE.Biliary Elimination of Low-Dose Methotrexate in Humans. Arthritis Rheum (1990) 33(6):898–902. doi: 10.1002/art.1780330620 2363742

[46] Schmiegelow K. Advances in Individual Prediction of Methotrexate Toxicity: A Review. Br J Haematol (2009) 146(5):489–503. doi: 10.1111/j.1365-2141.2009.07765.x 19538530

[47] Widemann BC Sung E Anderson L Salzer WL Balis FM Monitjo KS. Pharmacokinetics and Metabolism of the Methotrexate Metabolite 2, 4-Diamino-N(10)-Methylpteroic Acid. J Pharmacol Exp Ther (2000) 294(3):894–901. 10945838

[48] Larimer CM Slavnic D Pitstick LD Green JM. Comparison of Substrate Specificity of Escherichia Coli P-Aminobenzoyl-Glutamate Hydrolase With Pseudomonas Carboxypeptidase G. Adv Enzyme Res (2014) 2(1):39–48. doi: 10.4236/aer.2014.21004 27795973 PMC5082436

[49] Buchen S Ngampolo D Melton RG Hasan C Zoubek A Henze G. Carboxypeptidase G2 Rescue in Patients With Methotrexate Intoxication and Renal Failure. Br J Cancer (2005) 92(3):480–7. doi: 10.1038/sj.bjc.6602337 PMC2362096 15668713

[50] Svahn T Mellgren K Harila-Saari A Åsberg A Kanerva J Jónsson Ó. Delayed Elimination of High-Dose Methotrexate and Use of Carboxypeptidase G2 in Pediatric Patients During Treatment for Acute Lymphoblastic Leukemia. Pediatr Blood Cancer (2017) 64(7):1–7. doi: 10.1002/pbc.26395 27966809

[51] Chan ES Cronstein BN. Methotrexate–How Does It Really Work? Nat Rev Rheumatol (2010) 6(3):175–8. doi: 10.1038/nrrheum.2010.5 20197777

[52] Budzik GP Colletti LM Faltynek CR. Effects of Methotrexate on Nucleotide Pools in Normal Human T Cells and the CEM T Cell Line. Life Sci (2000) 66(23):2297–307. doi: 10.1016/s0024-3205(00)00559-2 10855951

[53] Morgan SL Baggott JE Vaughn WH Austin JS Veitch TA Lee JY. Supplementation With Folic Acid During Methotrexate Therapy for Rheumatoid Arthritis. A Double-Blind, Placebo-Controlled Trial. Ann Intern Med (1994) 121(11):833–41. doi: 10.7326/0003-4819-121-11-199412010-00002 7978695

[54] Morgan SL Baggott JE Vaughn WH Young PK Austin JV Krumdieck CL. The Effect of Folic Acid Supplementation on the Toxicity of Low-Dose Methotrexate in Patients With Rheumatoid Arthritis. Arthritis Rheum (1990) 33(1):9–18. doi: 10.1002/art.1780330102 2405864

[55] Shiroky JB Neville C Esdaile JM Choquette D Zummer M Hazeltine M. Low-Dose Methotrexate With Leucovorin (Folinic Acid) in the Management of Rheumatoid Arthritis. Results of a Multicenter Randomized, Double-Blind, Placebo-Controlled Trial. Arthritis Rheum (1993) 36(6):795–803. doi: 10.1002/art.1780360609 8507221

[56] Cronstein BN Sitkovsky M. Adenosine and Adenosine Receptors in the Pathogenesis and Treatment of Rheumatic Diseases. Nat Rev Rheumatol (2017) 13(1):41–51. doi: 10.1038/nrrheum.2016.178 27829671 PMC5173391

[57] Cronstein BN Naime D Ostad E. The Antiinflammatory Mechanism of Methotrexate. Increased Adenosine Release at Inflamed Sites Diminishes Leukocyte Accumulation in an *In Vivo* Model of Inflammation. J Clin Invest (1993) 92(6):2675–82. doi: 10.1172/jci116884 PMC288465 8254024

[58] Montesinos MC Yap JS Desai A Posadas I McCrary CT Cronstein BN. Reversal of the Antiinflammatory Effects of Methotrexate by the Nonselective Adenosine Receptor Antagonists Theophylline and Caffeine: Evidence That the Antiinflammatory Effects of Methotrexate Are Mediated *via* Multiple Adenosine Receptors in Rat Adjuvant Arthritis. Arthritis Rheum (2000) 43(3):656–63. doi: 10.1002/1529-0131(200003)43:3<656::aid-anr23>3.0.co;2-h 10728760

[59] Nesher G Mates M Zevin S. Effect of Caffeine Consumption on Efficacy of Methotrexate in Rheumatoid Arthritis. Arthritis Rheum (2003) 48(2):571–2. doi: 10.1002/art.10766 12571869

[60] Allard D Turcotte M Stagg J. Targeting A2 Adenosine Receptors in Cancer. Immunol Cell Biol (2017) 95(4):333–9. doi: 10.1038/icb.2017.8 28174424

[61] Montesinos MC Takedachi M Thompson LF Wilder TF Fernández P Cronstein BN. The Antiinflammatory Mechanism of Methotrexate Depends on Extracellular Conversion of Adenine Nucleotides to Adenosine by Ecto-5'-Nucleotidase: Findings in a Study of Ecto-5'-Nucleotidase Gene-Deficient Mice. Arthritis Rheum (2007) 56(5):1440–5. doi: 10.1002/art.22643 17469101

[62] Montesinos MC Desai A Cronstein BN. Suppression of Inflammation by Low-Dose Methotrexate Is Mediated by Adenosine A2A Receptor But Not A3 Receptor Activation in Thioglycollate-Induced Peritonitis. Arthritis Res Ther (2006) 8(2):R53. doi: 10.1186/ar1914 16519795 PMC1526598

[63] Montesinos MC Desai A Delano D Chen JF Fink JS Jacobson MA. Adenosine A2A or A3 Receptors Are Required for Inhibition of Inflammation by Methotrexate and Its Analog MX-68. Arthritis Rheum (2003) 48(1):240–7. doi: 10.1002/art.10712 12528125

[64] Peres RS Liew FY Talbot J Carregaro V Oliveira RD Almeida SL. Low Expression of CD39 on Regulatory T Cells as a Biomarker for Resistance to Methotrexate Therapy in Rheumatoid Arthritis. Proc Natl Acad Sci USA (2015) 112(8):2509–14. doi: 10.1073/pnas.1424792112 PMC4345589 25675517

[65] Chalupsky K Cai H. Endothelial Dihydrofolate Reductase: Critical for Nitric Oxide Bioavailability and Role in Angiotensin II Uncoupling of Endothelial Nitric Oxide Synthase. Proc Natl Acad Sci USA (2005) 102(25):9056–61. doi: 10.1073/pnas.0409594102 PMC1157015 15941833

[66] Crabtree MJ Tatham AL Hale AB Alp NJ Channon KM. Critical Role for Tetrahydrobiopterin Recycling by Dihydrofolate Reductase in Regulation of Endothelial Nitric-Oxide Synthase Coupling: Relative Importance of the *De Novo* Biopterin Synthesis Versus Salvage Pathways. J Biol Chem (2009) 284(41):28128–36. doi: 10.1074/jbc.M109.041483 PMC2788863 19666465

[67] Sugiyama T Levy BD Michel T. Tetrahydrobiopterin Recycling, a Key Determinant of Endothelial Nitric-Oxide Synthase-Dependent Signaling Pathways in Cultured Vascular Endothelial Cells. J Biol Chem (2009) 284(19):12691–700. doi: 10.1074/jbc.M809295200 PMC2675998 19286667

[68] van den Berg WB. Anti-Cytokine Therapy in Chronic Destructive Arthritis. Arthritis Res (2001) 3(1):18–26. doi: 10.1186/ar136 11178124 PMC128880

[69] Chang DM Weinblatt ME Schur PH. The Effects of Methotrexate on Interleukin 1 in Patients With Rheumatoid Arthritis. J Rheumatol (1992) 19(11):1678–82. 1491385

[70] Gerards AH de Lathouder S de Groot ER Dijkmans BA Aarden LA. Inhibition of Cytokine Production by Methotrexate. Studies in Healthy Volunteers and Patients With Rheumatoid Arthritis. Rheumatol (Oxford) (2003) 42(10):1189–96. doi: 10.1093/rheumatology/keg323 12777636

[71] Rudwaleit M Yin Z Siegert S Grolms M Radbruch A Braun J. Response to Methotrexate in Early Rheumatoid Arthritis Is Associated With a Decrease of T Cell Derived Tumour Necrosis Factor Alpha, Increase of Interleukin 10, and Predicted by the Initial Concentration of Interleukin 4. Ann Rheum Dis (2000) 59(4):311–4. doi: 10.1136/ard.59.4.311 PMC1753104 10733482

[72] Chagoya de Sánchez V Hernández Múñoz R Suárez J Vidrio S Yáñez L Díaz Múñoz M. Day-Night Variations of Adenosine and Its Metabolizing Enzymes in the Brain Cortex of the Rat–Possible Physiological Significance for the Energetic Homeostasis and the Sleep-Wake Cycle. Brain Res (1993) 612(1-2):115–21. doi: 10.1016/0006-8993(93)91651-8 8330191

[73] Chagoya de Sánchez V. Circadian Variations of Adenosine and of Its Metabolism. Could Adenosine be a Molecular Oscillator for Circadian Rhythms? Can J Physiol Pharmacol (1995) 73(3):339–55. doi: 10.1139/y95-044 7648513

[74] Chagoya de Sánchez V Hernández-Muñoz R Suárez J Vidrio S Yáñez L Aguilar-Roblero R. Temporal Variations of Adenosine Metabolism in Human Blood. Chronobiol Int (1996) 13(3):163–77. doi: 10.3109/07420529609012650 8874980

[75] Merrill JT Shen C Schreibman D Coffey D Zakharenko O Fisher R. Adenosine A1 Receptor Promotion of Multinucleated Giant Cell Formation by Human Monocytes: A Mechanism for Methotrexate-Induced Nodulosis in Rheumatoid Arthritis. Arthritis Rheum (1997) 40(7):1308–15. doi: 10.1002/1529-0131(199707)40:7<1308::aid-art16>3.0.co;2-m 9214432

[76] Chan ES Montesinos MC Fernandez P Desai A Delano DL Yee H. Adenosine A(2A) Receptors Play a Role in the Pathogenesis of Hepatic Cirrhosis. Br J Pharmacol (2006) 148(8):1144–55. doi: 10.1038/sj.bjp.0706812 PMC1752015 16783407

[77] Che J Chan ES Cronstein BN. Adenosine A2A Receptor Occupancy Stimulates Collagen Expression by Hepatic Stellate Cells *via* Pathways Involving Protein Kinase A, Src, and Extracellular Signal-Regulated Kinases 1/2 Signaling Cascade or P38 Mitogen-Activated Protein Kinase Signaling Pathway. Mol Pharmacol (2007) 72(6):1626–36. doi: 10.1124/mol.107.038760 17872970

[78] Weisman MH Furst DE Park GS Kremer JM Smith KM Wallace DJ. Risk Genotypes in Folate-Dependent Enzymes and Their Association With Methotrexate-Related Side Effects in Rheumatoid Arthritis. Arthritis Rheum (2006) 54(2):607–12. doi: 10.1002/art.21573 16447238

[79] Kremer JM Alarcón GS Weinblatt ME Kaymakcian MV Macaluso M Cannon GW. Clinical, Laboratory, Radiographic, and Histopathologic Features of Methotrexate-Associated Lung Injury in Patients With Rheumatoid Arthritis: A Multicenter Study With Literature Review. Arthritis Rheum (1997) 40(10):1829–37. doi: 10.1002/art.1780401016 9336418

[80] Hsu PC Lan JL Hsieh TY Jan YJ Huang WN. Methotrexate Pneumonitis in a Patient With Rheumatoid Arthritis. J Microbiol Immunol Infect (2003) 36(2):137–40. 12886966

[81] Kim YJ Song M Ryu JC. Mechanisms Underlying Methotrexate-Induced Pulmonary Toxicity. Expert Opin Drug Saf (2009) 8(4):451–8. doi: 10.1517/14740330903066734 19538103

[82] Shahbaz M Kamran SH Anwar R. Amelioration of Bleomycin and Methotrexate-Induced Pulmonary Toxicity by Serratiopeptidase and Fisetin. Nutr Cancer (2020), 1–10. doi: 10.1080/01635581.2020.1860242 33353415

[83] Varone F Iovene B Sgalla G Calvello M Calabrese A Larici AR. Fibrotic Hypersensitivity Pneumonitis: Diagnosis and Management. Lung (2020) 198(3):429–40. doi: 10.1007/s00408-020-00360-3 32415523

[84] Virdee G Bleasdale J Ikramullah M Graham-Clarke E. Sertraline-Induced Hypersensitivity Pneumonitis. BMJ Case Rep (2019) 12(12):e230724. doi: 10.1136/bcr-2019-230724 PMC6936525 31862812

[85] Invernizzi R Wu BG Barnett J Ghai P Kingston S Hewitt RJ. The Respiratory Microbiome in Chronic Hypersensitivity Pneumonitis Is Distinct From That of Idiopathic Pulmonary Fibrosis. Am J Respir Crit Care Med (2021) 203(3):339–47. doi: 10.1164/rccm.202002-0460OC PMC7874329 32692582

[86] Strangfeld A Burmester GR. Methotrexate: What Are the True Risks of Treatment? Ann Rheum Dis (2020) 79(10):1267–8. doi: 10.1136/annrheumdis-2020-217207 32611600

[87] Mercer LK Askling J Raaschou P Dixon WG Dreyer L Hetland ML. Risk of Invasive Melanoma in Patients With Rheumatoid Arthritis Treated With Biologics: Results From a Collaborative Project of 11 European Biologic Registers. Ann Rheum Dis (2017) 76(2):386–91. doi: 10.1136/annrheumdis-2016-209285 PMC5284347 27307502

[88] Mercer LK Green AC Galloway JB Davies R Lunt M Dixon WG. The Influence of Anti-TNF Therapy Upon Incidence of Keratinocyte Skin Cancer in Patients With Rheumatoid Arthritis: Longitudinal Results From the British Society for Rheumatology Biologics Register. Ann Rheum Dis (2012) 71(6):869–74. doi: 10.1136/annrheumdis-2011-200622 PMC3371225 22241900

[89] Raaschou P Simard JF Asker Hagelberg C Askling J. Rheumatoid Arthritis, Anti-Tumour Necrosis Factor Treatment, and Risk of Squamous Cell and Basal Cell Skin Cancer: Cohort Study Based on Nationwide Prospectively Recorded Data From Sweden. BMJ (2016) 352i262. doi: 10.1136/bmj.i262 PMC4730989 26823527

[90] Plant D Maciejewski M Smith S Nair N Hyrich K Ziemek D. Profiling of Gene Expression Biomarkers as a Classifier of Methotrexate Nonresponse in Patients With Rheumatoid Arthritis. Arthritis Rheumatol (2019) 71(5):678–84. doi: 10.1002/art.40810 PMC9328381 30615300

[91] de Rotte M Pluijm SMF de Jong PHP Bulatović Ćalasan M Wulffraat NM Weel A. Development and Validation of a Prognostic Multivariable Model to Predict Insufficient Clinical Response to Methotrexate in Rheumatoid Arthritis. PLoS One (2018) 13(12):e0208534. doi: 10.1371/journal.pone.0208534 30532219 PMC6287811

[92] Sergeant JC Hyrich KL Anderson J Kopec-Harding K Hope HF Symmons DPM. Prediction of Primary Non-Response to Methotrexate Therapy Using Demographic, Clinical and Psychosocial Variables: Results From the UK Rheumatoid Arthritis Medication Study (RAMS). Arthritis Res Ther (2018) 20(1):147. doi: 10.1186/s13075-018-1645-5 30005689 PMC6044018

[93] Teitsma XM Jacobs JWG Welsing PMJ de Jong PHP Hazes JMW Weel A. Inadequate Response to Treat-to-Target Methotrexate Therapy in Patients With New-Onset Rheumatoid Arthritis: Development and Validation of Clinical Predictors. Ann Rheum Dis (2018) 77(9):1261–7. doi: 10.1136/annrheumdis-2018-213035 29760159

[94] Smolen JS Landewé RBM Bijlsma JWJ Burmester GR Dougados M Kerschbaumer A. EULAR Recommendations for the Management of Rheumatoid Arthritis With Synthetic and Biological Disease-Modifying Antirheumatic Drugs: 2019 Update. Ann Rheum Dis (2020) 79(6):685–99. doi: 10.1136/annrheumdis-2019-216655 31969328

[95] Halilova KI Brown EE Morgan SL Bridges SL Jr. Hwang MH Arnett DK. Markers of Treatment Response to Methotrexate in Rheumatoid Arthritis: Where Do We Stand? Int J Rheumatol (2012) 2012978396. doi: 10.1155/2012/978396 PMC3400362 22844292

[96] Scher JU Abramson SB. The Microbiome and Rheumatoid Arthritis. Nat Rev Rheumatol (2011) 7(10):569–78. doi: 10.1038/nrrheum.2011.121 PMC3275101 21862983

[97] Scher JU Sczesnak A Longman RS Segata N Ubeda C Bielski C. Expansion of Intestinal Prevotella Copri Correlates With Enhanced Susceptibility to Arthritis. Elife (2013) 2:e01202. doi: 10.7554/eLife.01202 24192039 PMC3816614

[98] Scher JU Ubeda C Artacho A Attur M Isaac S Reddy SM. Decreased Bacterial Diversity Characterizes the Altered Gut Microbiota in Patients With Psoriatic Arthritis, Resembling Dysbiosis in Inflammatory Bowel Disease. Arthritis Rheumatol (2015) 67(1):128–39. doi: 10.1002/art.38892 PMC4280348 25319745

[99] Sousa T Paterson R Moore V Carlsson A Abrahamsson B Basit AW. The Gastrointestinal Microbiota as a Site for the Biotransformation of Drugs. Int J Pharm (2008) 363(1-2):1–25. doi: 10.1016/j.ijpharm.2008.07.009 18682282

[100] Birer C Wright ES. Capturing the Complex Interplay Between Drugs and the Intestinal Microbiome. Clin Pharmacol Ther (2019) 106(3):501–4. doi: 10.1002/cpt.1505 PMC7574698 31102465

[101] Maini Rekdal V Bess EN Bisanz JE Turnbaugh PJ Balskus EP. Discovery and Inhibition of an Interspecies Gut Bacterial Pathway for Levodopa Metabolism. Science (2019) 364(6445):eaau6323. doi: 10.1126/science.aau6323 31196984 PMC7745125

[102] Angelis-Stoforidis P Vajda FJ. Christophidis N.Methotrexate Polyglutamate Levels in Circulating Erythrocytes and Polymorphs Correlate With Clinical Efficacy in Rheumatoid Arthritis. Clin Exp Rheumatol (1999) 17(3):313–20. 10410264

[103] Dervieux T Furst D Lein DO Capps R Smith K Walsh M. Polyglutamation of Methotrexate With Common Polymorphisms in Reduced Folate Carrier, Aminoimidazole Carboxamide Ribonucleotide Transformylase, and Thymidylate Synthase Are Associated With Methotrexate Effects in Rheumatoid Arthritis. Arthritis Rheum (2004) 50(9):2766–74. doi: 10.1002/art.20460 15457444

[104] Danila MI Hughes LB Brown EE Morgan SL Baggott JE Arnett DK. Measurement of Erythrocyte Methotrexate Polyglutamate Levels: Ready for Clinical Use in Rheumatoid Arthritis? Curr Rheumatol Rep (2010) 12(5):342–7. doi: 10.1007/s11926-010-0120-3 PMC3769795 20665136

[105] Stamp LK O'Donnell JL Chapman PT Zhang M James J Frampton C. Methotrexate Polyglutamate Concentrations Are Not Associated With Disease Control in Rheumatoid Arthritis Patients Receiving Long-Term Methotrexate Therapy. Arthritis Rheum (2010) 62(2):359–68. doi: 10.1002/art.27201 20112376

[106] Lebbe C Beyeler C Gerber NJ Reichen J. Intraindividual Variability of the Bioavailability of Low Dose Methotrexate After Oral Administration in Rheumatoid Arthritis. Ann Rheum Dis (1994) 53(7):475–7. doi: 10.1136/ard.53.7.475 PMC1005374 7944622

[107] Hornung N Ellingsen T Attermann J Stengaard-Pedersen K Poulsen JH. Patients With Rheumatoid Arthritis Treated With Methotrexate (MTX): Concentrations of Steady-State Erythrocyte MTX Correlate to Plasma Concentrations and Clinical Efficacy. J Rheumatol (2008) 35(9):1709–15. 18634162

[108] Bluett J Sergeant JC MacGregor AJ Chipping JR Marshall T Symmons DPM. Risk Factors for Oral Methotrexate Failure in Patients With Inflammatory Polyarthritis: Results From a UK Prospective Cohort Study. Arthritis Res Ther (2018) 20(1):50. doi: 10.1186/s13075-018-1544-9 29554956 PMC5859656

[109] Dekkers JS Bergstra SA Chopra A Tikly M Fonseca JE Salomon-Escoto K. Autoantibody Status Is Not Associated With Early Treatment Response to First-Line Methotrexate in Patients With Early Rheumatoid Arthritis. Rheumatol (Oxford) (2019) 58(1):149–53. doi: 10.1093/rheumatology/key263 30204896

[110] Hider SL Silman AJ Thomson W Lunt M Bunn D Symmons DP. Can Clinical Factors at Presentation be Used to Predict Outcome of Treatment With Methotrexate in Patients With Early Inflammatory Polyarthritis? Ann Rheum Dis (2009) 68(1):57–62. doi: 10.1136/ard.2008.088237 18292102 PMC2596302

[111] Gupta V Katiyar S Singh A Misra R Aggarwal A. CD39 Positive Regulatory T Cell Frequency as a Biomarker of Treatment Response to Methotrexate in Rheumatoid Arthritis. Int J Rheum Dis (2018) 21(8):1548–56. doi: 10.1111/1756-185x.13333 30146748

[112] López-Rodríguez R Ferreiro-Iglesias A Lima A Bernardes M Pawlik A Paradowska-Gorycka A. Replication Study of Polymorphisms Associated With Response to Methotrexate in Patients With Rheumatoid Arthritis. Sci Rep (2018) 8(1):7342. doi: 10.1038/s41598-018-25634-y 29743634 PMC5943457

[113] Wessels JA van der Kooij SM le Cessie S Kievit W Barerra P Allaart CF. A Clinical Pharmacogenetic Model to Predict the Efficacy of Methotrexate Monotherapy in Recent-Onset Rheumatoid Arthritis. Arthritis Rheum (2007) 56(6):1765–75. doi: 10.1002/art.22640 17530705

[114] Eektimmerman F Allaart CF Hazes JM Madhar MB den Broeder AA Fransen J. Validation of a Clinical Pharmacogenetic Model to Predict Methotrexate Nonresponse in Rheumatoid Arthritis Patients. Pharmacogenomics (2019) 20(2):85–93. doi: 10.2217/pgs-2018-0144 30628539

[115] Jenko B Tomšič M Jekić B Milić V Dolžan V. Praprotnik S.Clinical Pharmacogenetic Models of Treatment Response to Methotrexate Monotherapy in Slovenian and Serbian Rheumatoid Arthritis Patients: Differences in Patient's Management May Preclude Generalization of the Models. Front Pharmacol (2018) 920:20. doi: 10.3389/fphar.2018.00020 PMC5788961 29422864

[116] López-Rodríguez R Ferreiro-Iglesias A Lima A Bernardes M Pawlik A Paradowska-Gorycka A. Evaluation of a Clinical Pharmacogenetics Model to Predict Methotrexate Response in Patients With Rheumatoid Arthritis. Pharmacogenomics J (2018) 18(4):539–45. doi: 10.1038/s41397-018-0017-5 29520081

[117] Routy B Le Chatelier E Derosa L Duong CPM Alou MT Daillère R. Gut Microbiome Influences Efficacy of PD-1-Based Immunotherapy Against Epithelial Tumors. Science (2018) 359(6371):91–7. doi: 10.1126/science.aan3706 29097494

[118] Matson V Fessler J Bao R Chongsuwat T Zha Y Alegre ML. The Commensal Microbiome Is Associated With Anti-PD-1 Efficacy in Metastatic Melanoma Patients. Science (2018) 359(6371):104–8. doi: 10.1126/science.aao3290 PMC6707353 29302014

[119] Gopalakrishnan V Spencer CN Nezi L Reuben A Andrews MC Karpinets TV. Gut Microbiome Modulates Response to Anti-PD-1 Immunotherapy in Melanoma Patients. Science (2018) 359(6371):97–103. doi: 10.1126/science.aan4236 29097493 PMC5827966

[120] Spanogiannopoulos P Bess EN Carmody RN Turnbaugh PJ. The Microbial Pharmacists Within Us: A Metagenomic View of Xenobiotic Metabolism. Nat Rev Microbiol (2016) 14(5):273–87. doi: 10.1038/nrmicro.2016.17 PMC5243131 26972811

[121] Koppel N Maini Rekdal V Balskus EP. Chemical Transformation of Xenobiotics by the Human Gut Microbiota. Science (2017) 356(6344):eaag2770. doi: 10.1126/science.aag2770 28642381 PMC5534341

[122] Zimmermann M Zimmermann-Kogadeeva M Wegmann R Goodman AL. Mapping Human Microbiome Drug Metabolism by Gut Bacteria and Their Genes. Nature (2019) 570(7762):462–7. doi: 10.1038/s41586-019-1291-3 PMC6597290 31158845

[123] Zhang X Zhang D Jia H Feng Q Wang D Liang D. The Oral and Gut Microbiomes Are Perturbed in Rheumatoid Arthritis and Partly Normalized After Treatment. Nat Med (2015) 21(8):895–905. doi: 10.1038/nm.3914 26214836

[124] Artacho A Isaac S Nayak R Flor-Duro A Alexander M Koo I. The Pretreatment Gut Microbiome Is Associated With Lack of Response to Methotrexate in New-Onset Rheumatoid Arthritis. Arthritis Rheumatol (2020) 73(6):931-42. doi: 10.1002/art.41622 PMC11293279 33314800

[125] Refsum H Christensen B Djurhuus R Ueland PM. Interaction Between Methotrexate, "Rescue" Agents and Cell Proliferation as Modulators of Homocysteine Export From Cells in Culture. J Pharmacol Exp Ther (1991) 258(2):559–66. 1865358

[126] Howell SB Mansfield SJ Taetle R. Thymidine and Hypoxanthine Requirements of Normal and Malignant Human Cells for Protection Against Methotrexate Cytotoxicity. Cancer Res (1981) 41(3):945–50. 6257387

[127] Qiu Q Huang J Shu X Fan H Zhou Y Xiao C. Polymorphisms and Pharmacogenomics for the Clinical Efficacy of Methotrexate in Patients With Rheumatoid Arthritis: A Systematic Review and Meta-Analysis. Sci Rep (2017) 7(44015):1–24. doi: 10.1038/srep44015 28266606 PMC5339794

[128] Pannu AK. Methotrexate Overdose in Clinical Practice. Curr Drug Metab (2019) 20(9):714–9. doi: 10.2174/1389200220666190806140844 31385765

[129] Widemann BC Adamson PC. Understanding and Managing Methotrexate Nephrotoxicity. Oncologist (2006) 11(6):694–703. doi: 10.1634/theoncologist.11-6-694 16794248

[130] Katchamart W Trudeau J Phumethum V Bombardier C. Efficacy and Toxicity of Methotrexate (MTX) Monotherapy Versus MTX Combination Therapy With Non-Biological Disease-Modifying Antirheumatic Drugs in Rheumatoid Arthritis: A Systematic Review and Meta-Analysis. Ann Rheum Dis (2009) 68(7):1105–12. doi: 10.1136/ard.2008.099861 PMC2689526 19054823

[131] El-Sheikh AA Morsy MA Hamouda AH. Protective Mechanisms of Thymoquinone on Methotrexate-Induced Intestinal Toxicity in Rats. Pharmacogn Mag (2016) 12(Suppl 1):S76–81. doi: 10.4103/0973-1296.176106 PMC4792005 27041864

[132] Keefe DM Schubert MM Elting LS Sonis ST Epstein JB Raber-Durlacher JE. Updated Clinical Practice Guidelines for the Prevention and Treatment of Mucositis. Cancer (2007) 109(5):820–31. doi: 10.1002/cncr.22484 17236223

[133] Touchefeu Y Montassier E Nieman K Gastinne T Potel G Bruley des Varannes S. Systematic Review: The Role of the Gut Microbiota in Chemotherapy- or Radiation-Induced Gastrointestinal Mucositis - Current Evidence and Potential Clinical Applications. Aliment Pharmacol Ther (2014) 40(5):409–21. doi: 10.1111/apt.12878 25040088

[134] Sukhotnik I Pollak Y Coran AG Pilatov J Bejar J Mogilner JG. Glutamine Attenuates the Inhibitory Effect of Methotrexate on TLR Signaling During Intestinal Chemotherapy-Induced Mucositis in a Rat. Nutr Metab (Lond) (2014) 11(1):17–27. doi: 10.1186/1743-7075-11-17 24742067 PMC4005622

[135] Cetiner M Sener G Sehirli AO Ekşioğlu-Demiralp E Ercan F Sirvanci S. Taurine Protects Against Methotrexate-Induced Toxicity and Inhibits Leukocyte Death. Toxicol Appl Pharmacol (2005) 209(1):39–50. doi: 10.1016/j.taap.2005.03.009 15890378

[136] Higuchi T Yoshimura M Oka S Tanaka K Naito T Yuhara S. Modulation of Methotrexate-Induced Intestinal Mucosal Injury by Dietary Factors. Hum Exp Toxicol (2020) 39(4):500–13. doi: 10.1177/0960327119896605 31876189

[137] Manichanh C Rigottier-Gois L Bonnaud E Gloux K Pelletier E Frangeul L. Reduced Diversity of Faecal Microbiota in Crohn's Disease Revealed by a Metagenomic Approach. Gut (2006) 55(2):205–11. doi: 10.1136/gut.2005.073817 PMC1856500 16188921

[138] Nishikawa J Kudo T Sakata S Benno Y Sugiyama T. Diversity of Mucosa-Associated Microbiota in Active and Inactive Ulcerative Colitis. Scand J Gastroenterol (2009) 44(2):180–6. doi: 10.1080/00365520802433231 18825588

[139] Huang X Fang Q Rao T Zhou L Zeng X Tan Z. Leucovorin Ameliorated Methotrexate Induced Intestinal Toxicity *via* Modulation of the Gut Microbiota. Toxicol Appl Pharmacol (2020) 391:114900. doi: 10.1016/j.taap.2020.114900 32061593

[140] Wang Y Wiesnoski DH Helmink BA Gopalakrishnan V Choi K DuPont HL. Fecal Microbiota Transplantation for Refractory Immune Checkpoint Inhibitor-Associated Colitis. Nat Med (2018) 24(12):1804–8. doi: 10.1038/s41591-018-0238-9 PMC6322556 30420754

[141] Kragsnaes MS Kjeldsen J Horn HC Munk HL Pedersen JK Just SA. Safety and Efficacy of Faecal Microbiota Transplantation for Active Peripheral Psoriatic Arthritis: An Exploratory Randomised Placebo-Controlled Trial. Ann Rheum Dis (2021) 80(9):1158–67. doi: 10.1136/annrheumdis-2020-219511 33926922

[142] Isaac S Scher JU Djukovic A Jiménez N Littman DR Abramson SB. Short- and Long-Term Effects of Oral Vancomycin on the Human Intestinal Microbiota. J Antimicrob Chemother (2017) 72(1):128–36. doi: 10.1093/jac/dkw383 PMC5161046 27707993

[143] Maier L Pruteanu M Kuhn M Zeller G Telzerow A Anderson EE. Extensive Impact of Non-Antibiotic Drugs on Human Gut Bacteria. Nature (2018) 555(7698):623–8. doi: 10.1038/nature25979 PMC6108420 29555994

[144] Chen J Wright K Davis JM Jeraldo P Marietta EV Murray J. An Expansion of Rare Lineage Intestinal Microbes Characterizes Rheumatoid Arthritis. Genome Med (2016) 8(1):43. doi: 10.1186/s13073-016-0299-7 27102666 PMC4840970

[145] Fijlstra M Ferdous M Koning AM Rings EH Harmsen HJ Tissing WJ. Substantial Decreases in the Number and Diversity of Microbiota During Chemotherapy-Induced Gastrointestinal Mucositis in a Rat Model. Support Care Cancer (2015) 23(6):1513–22. doi: 10.1007/s00520-014-2487-6 25376667

[146] Filman DJ Bolin JT Matthews DA Kraut J. Crystal Structures of Escherichia Coli and Lactobacillus Casei Dihydrofolate Reductase Refined at 1.7 A Resolution. II. Environment of Bound NADPH and Implications for Catalysis. J Biol Chem (1982) 257(22):13663–72. 6815179

[147] Nayak RR Alexander M Deshpande I Stapleton-Gray K Rimal B Patterson AD. Methotrexate Impacts Conserved Pathways in Diverse Human Gut Bacteria Leading to Decreased Host Immune Activation. Cell Host Microbe (2021) 29(3):362–77.e11. doi: 10.1016/j.chom.2020.12.008 33440172 PMC7954989

[148] Hu N Liu X Mu Q Yu M Wang H Jiang Y. The Gut Microbiota Contributes to the Modulation of Intestinal CYP3A1 and P-Gp in Streptozotocin-Induced Type 1 Diabetic Rats. Eur J Pharm Sci (2021) 162:105833. doi: 10.1016/j.ejps.2021.105833 33826935

[149] Kragsnaes MS Kjeldsen J Horn HC Munk HL Pedersen FM Holt HM. Efficacy and Safety of Faecal Microbiota Transplantation in Patients With Psoriatic Arthritis: Protocol for a 6-Month, Double-Blind, Randomised, Placebo-Controlled Trial. BMJ Open (2018) 8(4):e019231. doi: 10.1136/bmjopen-2017-019231 PMC5922473 29703851

[150] Gjorevski N Ranga A Lutolf MP. Bioengineering Approaches to Guide Stem Cell-Based Organogenesis. Development (2014) 141(9):1794–804. doi: 10.1242/dev.101048 24757002

[151] Bhatia SN Ingber DE. Microfluidic Organs-on-Chips. Nat Biotechnol (2014) 32(8):760–72. doi: 10.1038/nbt.2989 25093883

[152] Lagier JC Khelaifia S Alou MT Ndongo S Dione N Hugon P. Culture of Previously Uncultured Members of the Human Gut Microbiota by Culturomics. Nat Microbiol (2016) 1(16203):1–8. doi: 10.1038/nmicrobiol.2016.203 PMC12094094 27819657

